# Extraction, Phytochemical Profiling, and Computational Evaluation of *Corymbia citriodora* Essential Oil as a COX-2 Inhibitor: Steam vs. Microwave-Assisted Distillation, GC–MS/MS, Docking, DFT, and MD Simulations

**DOI:** 10.3390/ph19091382

**Published:** 2026-09-01

**Authors:** Sabrina Koribeche, Sadjia Bertouche, Nassila Sabba, Naima Sahraoui, Farah Djelti, Faisal K. Alkholifi, Rana M. Al-dossari, Mohamed Said Kahaleras, Mostefa Hani, Yazid Chetbani, Yacine Karmi, Samia Daoudi-Hacini

**Affiliations:** 1 Laboratory of Matter’s Valorization and Recycling for Sustainable Development, Faculty of Mechanical and Process Engineering, University of Science and Technology Houari Boumediene (USTHB), Algiers 16111, Algeria; skoribeche@usthb.dz (S.K.); sbertouche@usthb.dz (S.B.); nsabba@usthb.dz (N.S.); sahraouinaima65@yahoo.fr (N.S.); 2 CancerLab N°30 Research Laboratory, Faculty of Natural Sciences, University of Tlemcen, Tlemcen 13000, Algeria; farah.djelti@univ-tlemcen.dz; 3 Department of Pharmacology & Toxicology, College of Pharmacy, Prince Sattam Bin Abdulaziz University, Al-Kharj 11942, Saudi Arabia; r.alsaffar@psau.edu.sa; 4 Laboratory of Energy Systems Technologies (LTSE), National Higher School of Technology and Engineering, Annaba 23005, Algeria; m.kahaleras@ensti-annaba.dz; 5 Faculty of Sciences and Technology, University of Jijel, Jijel 18000, Algeria; mostefa.hani@univ-jijel.dz; 6 Scientific and Technical Research Center for Physico-Chemical Analyses, Tipaza 42004, Algeria; chetbani.yazid92@gmail.com; 7 Electromechanical Department, Institute of Science and Applied Technics, University of Constantine 1, Constantine 25017, Algeria; yacine.karmi@umc.edu.dz; 8 Laboratory of Integrative Improvement of Plant Production (L-AIPV C2711100), Department of Agricultural and Forest Zoology, National Higher School of Agronomy (E.N.S.A), El Harrach, Algiers 16000, Algeria; samdaoudi25@gmail.com

**Keywords:** *Corymbia citriodora*, essential oil, molecular dynamics, density functional theory (DFT), MM/PBSA, ADMET profiling, computer-aided drug design, cyclooxygenase-2 (COX-2) inhibitors

## Abstract

**Background/Objectives:** Chronic inflammation sustained by cyclooxygenase-2 (COX-2) and inducible nitric oxide synthase (iNOS) underlies many human diseases, while the cardiovascular liabilities of coxibs have renewed interest in plant-derived alternatives. This study characterised the volatile composition of *Corymbia citriodora* essential oil under two distillation regimes and identified constituents able to inhibit human COX-2 and iNOS. **Methods:** Oils obtained by steam distillation (SD) and microwave-assisted steam distillation (MSD) were profiled by GC–MS/MS. All identified constituents were screened with SASA Vina against human COX-2 (PDB: 5KIR, 3LN1), ovine COX-1 (4COX) and human iNOS (3E7G), with native-ligand re-docking validating each protocol (RMSD 0.39–0.84 Å). The lead compound underwent density functional theory (B3LYP/3-21G/CPCM), 100 ns all-atom molecular dynamics, MM/GBSA and MM/PBSA decomposition, and pkCSM ADMET prediction. **Results:** Sixty-three constituents were identified (99.85% SD; 99.97% MSD). MSD enriched oxygenated monoterpenes (93.23% versus 88.27%), citronellal rose from 38.24% to 48.41%. (Z,Z,Z)-1,5,9,9-Tetramethyl-1,4,7-cycloundecatriene ranked highest at COX-2 (−8.0 to −8.2 kcal/mol) and also bound COX-1 (−8.8 kcal/mol) and iNOS (−6.8 kcal/mol). DFT indicated high kinetic stability (ΔE = 6.12 eV; η = 3.06 eV) and purely non-covalent hydrophobic binding. The COX-2 complex remained stable over 100 ns (Cα RMSD 0.17 ± 0.02 nm), with MM/GBSA and MM/PBSA binding energies of −23.98 and −21.95 kcal/mol and Val523 as the principal hotspot. ADMET prediction returned 96.61% human intestinal absorption and no mutagenicity, hepatotoxicity or hERG I blockade. **Conclusions:** MSD offers a faster route to a citronellal-enriched oil, and *C. citriodora* hydrocarbon sesquiterpenes emerge as chemically stable, multi-target COX-2/iNOS scaffolds. Comparable binding at COX-1 indicates that isoform selectivity remains to be established; in vitro enzymatic and cell-based validation is therefore required.

## 1. Introduction

Although inflammation serves as an indispensable, tightly regulated biological defense mechanism against tissue injury and pathogens, its chronic dysregulation constitutes the pathological cornerstone for a myriad of severe human diseases [1,2]. Conditions ranging from rheumatoid arthritis and neurodegenerative disorders to cardiovascular diseases and neoplastic transformations are all intimately linked to sustained inflammatory cascades [1,2,3]. At the molecular apex of this inflammatory cascade lies the cyclooxygenase (COX) enzyme system, which acts as the primary catalyst for the rate-limiting bisoxygenation of arachidonic acid into prostaglandin H_2_ (PGH_2_) [4,5,6]. PGH_2_ subsequently serves as the critical precursor for various hyperalgesic and pro-inflammatory prostanoids, including PGE_2_, PGI_2_, PGD_2_, PGF_2_α, and thromboxane A_2_ (TXA_2_) [5,6]. The COX-1 isoform is expressed constitutively in nearly all tissues, where it carries out vital homeostatic functions including preservation of the gastric mucosal lining, regulation of kidney activity, and the synthesis of thromboxane within platelets [4,5,7,8]. Conversely, the COX-2 isoform is highly inducible, rapidly upregulated by cytokines, mitogens, and growth factors during localized inflammatory and neoplastic responses [4,5,6]. Furthermore, in pro-inflammatory microenvironments, COX-2 frequently operates in tandem with inducible nitric oxide synthase (iNOS), creating a synergistic pathway where overproduced prostaglandin E_2_ and nitric oxide jointly amplify pain, edema, and tissue damage. Consequently, targeting these inducible inflammatory enzymes particularly through selective COX-2 inhibition has long been a primary objective in anti-inflammatory pharmacology [4,5,6].

The advent of targeted COX-2 antagonists, colloquially known as coxibs (e.g., rofecoxib and celecoxib), revolutionized pain management by drastically reducing the severe gastrointestinal toxicities associated with traditional, non-selective non-steroidal anti-inflammatory drugs (NSAIDs) [4,9,10]. However, the initial clinical optimism was soon eclipsed by alarming post-marketing and trial data demonstrating an increased incidence of thrombotic cardiovascular events with several coxibs [10,11,12]. Prolonged COX-2-specific inhibition was found to markedly suppress the production of vasoprotective and anti-aggregatory endothelial prostacyclin (PGI_2_), without simultaneously inhibiting the pro-thrombotic TXA_2_ generated by COX-1 in platelets, thereby shifting the prostanoid balance toward thrombosis [6,12,13]. This biochemical imbalance precipitates a pro-thrombotic state, associated with elevated risks of myocardial infarction and stroke, as shown in randomized trials and observational studies of rofecoxib and other coxibs [11,12,13]. The subsequent global market withdrawal of rofecoxib and the imposition of stringent boxed cardiovascular warnings on remaining coxibs created a major therapeutic dilemma in long-term anti-inflammatory management [9,10,11]. This clinical crisis has helped fuel a renaissance in natural product drug discovery, directing attention to plant-derived compounds as potentially safer anti-inflammatory agents targeting chronic inflammation-driven diseases [2,3,14].

Within land-dwelling plant species, *Corymbia citriodora* (Hook.) K.D. Hill & L.A.S. Johnson previously known under the taxonomic designation *Eucalyptus citriodora* Hook. is a tall member of the Myrtaceae family, indigenous to northeastern Australia and extensively cultivated throughout tropical and Mediterranean climates; this species is particularly noteworthy for its substantial ethnopharmacological significance [14,15,16]. Cultivated extensively for its highly fragrant, lemon-scented foliage, the essential oil extracted from its leaves exhibits a well-documented spectrum of bioactivities, including antimicrobial, antioxidant, and insecticidal effects, and has been traditionally used as an antipyretic, analgesic, and anti-inflammatory remedy [15,16]. Chemically, *C. citriodora* essential oil is typically rich in oxygenated monoterpenes such as citronellal, citronellol, and related derivatives, which contribute to strong radical-scavenging and antimicrobial activities in in vitro assays [15,16,17]. Historically, the macroscopic therapeutic efficacy of this citronellal-dominated chemotype has been supported by traditional use and preliminary in vivo or in vitro models, yet the precise molecular dynamics dictating how its diverse phytochemical constituents interact with human inflammatory targets remain largely elusive [2,15]. Essential oils are immensely complex biochemical matrices, and pharmacological responses are often driven not only by the most abundant monoterpenes but also by minor terpenoids and other constituents acting synergistically [2,14,15].

Despite the widespread pharmacological and agrochemical exploration of *C. citriodora* essential oils, a critical void persists in the current literature regarding the exact atomic-level mechanisms responsible for their anti-inflammatory efficacy, particularly at cyclooxygenase and inducible nitric oxide synthase targets. Existing work largely evaluates crude, unfractionated oils in phenotypic antimicrobial or antioxidant assays, or focuses on compositional profiling, rather than detailed interaction with COX and iNOS isoforms [15,16,17]. Where molecular modeling has been attempted for plant constituents more generally, approaches frequently rely on static docking and do not incorporate dynamic solvation, conformational sampling, or quantum-mechanical descriptions of electronic reactivity within the active site [18]. There is essentially no continuous multiscale framework in the available *C. citriodora* literature capable of simultaneously capturing ligand electronic structure, spatiotemporal conformational stability under physiological hydration, and rigorous endpoint thermodynamic binding free energies for specific constituents at COX-2 [14,15,16]. In the absence of such high-resolution biophysical mapping, the rational identification, optimization, and repurposing of individual *C. citriodora* phytochemicals into standardized, mechanism-guided anti-inflammatory leads remains out of reach, and current applications necessarily rely on empirical use of complex extracts rather than structure-based drug design [2,14,15].

To the best of our knowledge, the present study represents the first comprehensive, multiscale computational and phytochemical integration applied to *C. citriodora* essential oil across a multi-target inflammatory panel. The originality of this work lies in its dual-extraction approach and its advanced computer-aided drug design (CADD) pipeline. We directly juxtapose conventional steam distillation (SD) with microwave-assisted steam distillation (MSD) to assess extraction-driven phytochemical variability. Subsequently, we deploy a theoretical workflow that bridges classical mechanics with quantum chemistry across human COX-2 (PDB IDs: 5KIR and 3LN1), COX-1 (PDB ID: 4COX), and iNOS (PDB ID: 3E7G). Rather than relying on static docking alone, this study utilizes Density Functional Theory (DFT) at the B3LYP/3-21G level to map the intrinsic quantum electronic landscape and chemical hardness of the top-ranked candidate compounds. This is coupled with extensive 100 ns all-atom molecular dynamics (MD) simulations, Principal Component Analysis (PCA), Free Energy Landscape (FEL) mapping, and rigorous MM/PBSA and MM/GBSA thermodynamic decomposition, providing an atomistic reconstruction of the xenobiotic–receptor interface.

The overarching aim of this research is to systematically dissect the molecular basis of *C. citriodora*’s anti-inflammatory potential and isolate highly selective, chemically stable, and non-toxic COX-2/iNOS modulators from its volatile matrix. To achieve this, the specific objectives are threefold: To provide a comparative, high-resolution GC–MS/MS phytochemical fingerprint of the essential oil yielded by both SD and MSD techniques; to execute a high-throughput structure-based virtual screening of the entire identified chemical library (sixty-three constituents) against a multi-target panel comprising human COX-2 (5KIR and 3LN1), ovine COX-1 (4COX), and human iNOS (3E7G) to isolate the most potent scaffolds and assess isoform selectivity; and to rigorously characterize the top candidate scaffolds specifically focusing on the hydrocarbon sesquiterpene (Z,Z,Z)-1,5,9,9-tetramethyl-1,4,7-cycloundecatriene through quantum electronic profiling (DFT), multiscale molecular dynamics, and free energy thermodynamic decomposition, complemented by comprehensive predictive ADMET profiling.

Through this integrative framework, we aim to deliver a rational, residue-resolved blueprint for the development of next-generation, plant-derived selective COX-2 modulators.

## 2. Results and Discussion

### 2.1. Phytochemical Profiling of C. citriodora Essential Oil

**Figure 1:**
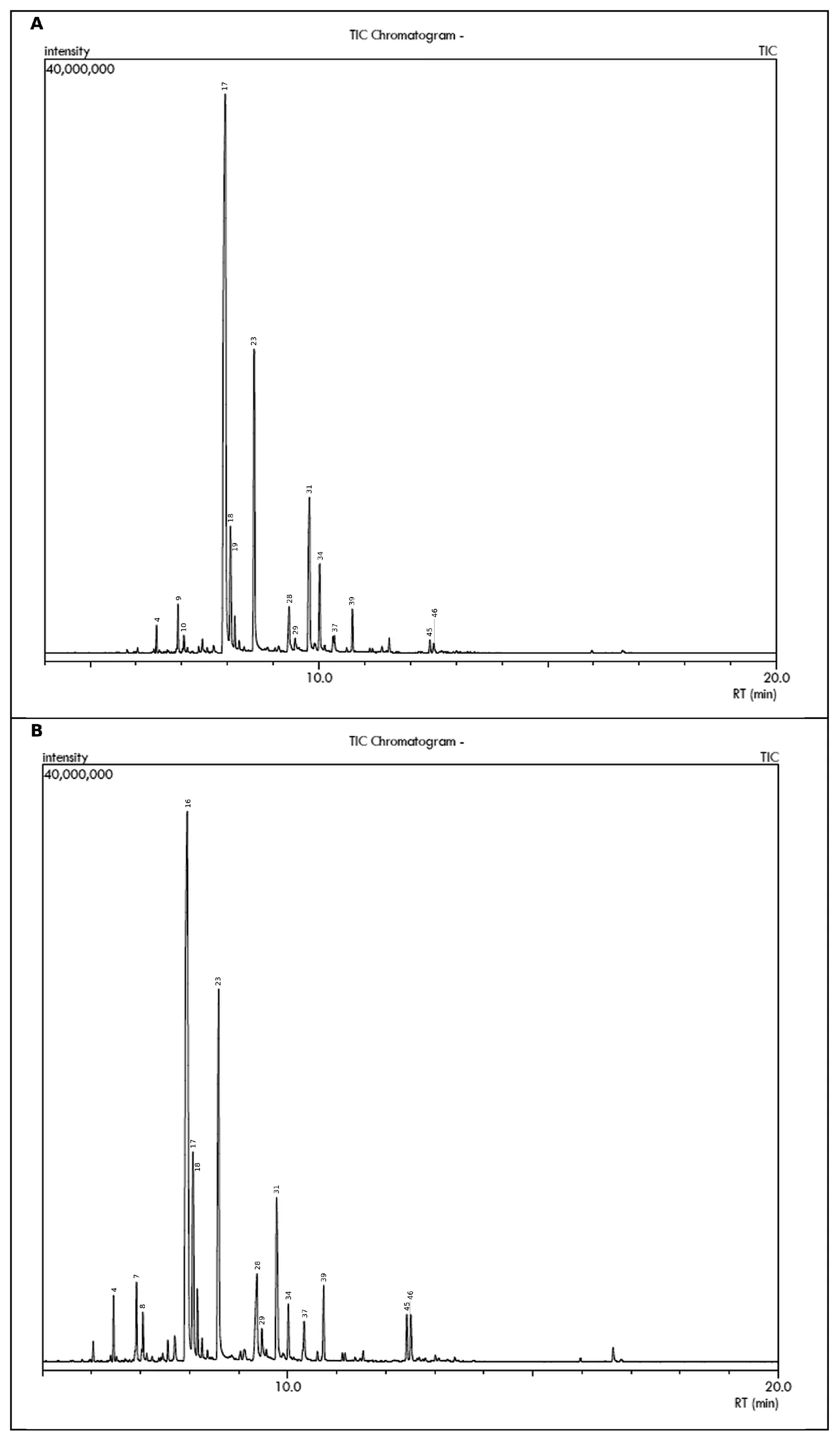
Total ion current (TIC) gas chromatography–mass spectrometry (GC–MS) chromatograms of *Corymbia citriodora* essential oil extracted by two different distillation techniques: (**A**) Microwave-Assisted Steam Distillation (MSD) and (**B**) Conventional Steam Distillation (SD). Peak numbers correspond to the volatile compounds identified in Table 1, with the major peak representing citronellal (RT≈7.95 min).

GC–MS-MS profiling of the essential oils obtained by SD and MSD allowed the unambiguous identification of sixty-three constituents accounting for 99.85% and 99.97% of the total composition, respectively (Table 1, Figure 1). Compound assignments relied on mass-spectral matching combined with Kovats retention indices, following current recommendations for the analysis and authentication of essential-oil volatiles [19,20]. The two extraction modalities yielded qualitatively comparable but quantitatively distinct chemical fingerprints, with MSD producing a marked enrichment of oxygenated monoterpenes (93.23%) relative to SD (88.27%), a contrast of the kind routinely resolved by comparative GC–MS profiling of volatile fractions [21]. Citronellal (CID 7794) was the dominant constituent in both samples, increasing from 38.24% (SD) to 48.41% (MSD), followed by citronellol (15.43%/15.16%), isopulegol (8.56%/6.46%), citronellyl isovalerate (7.04%/9.19%), and citronellic acid (5.00%/2.50%). Minor classes included hydrocarbon sesquiterpenes (2.66%/2.34%), hydrocarbon monoterpenes (2.58%/1.39%), oxygenated sesquiterpenes (3.72%/1.13%), and esters (0.05%/0.23%). The selective concentration of polar oxygenated terpenoids under microwave irradiation is consistent with the more efficient dielectric heating of these constituents and supports the use of MSD as a greener and more selective recovery technique for the citronellal chemotype.

### 2.2. Docking Protocol

#### 2.2.1. Docking Validation and RMSD Calculation

**Figure 2:**
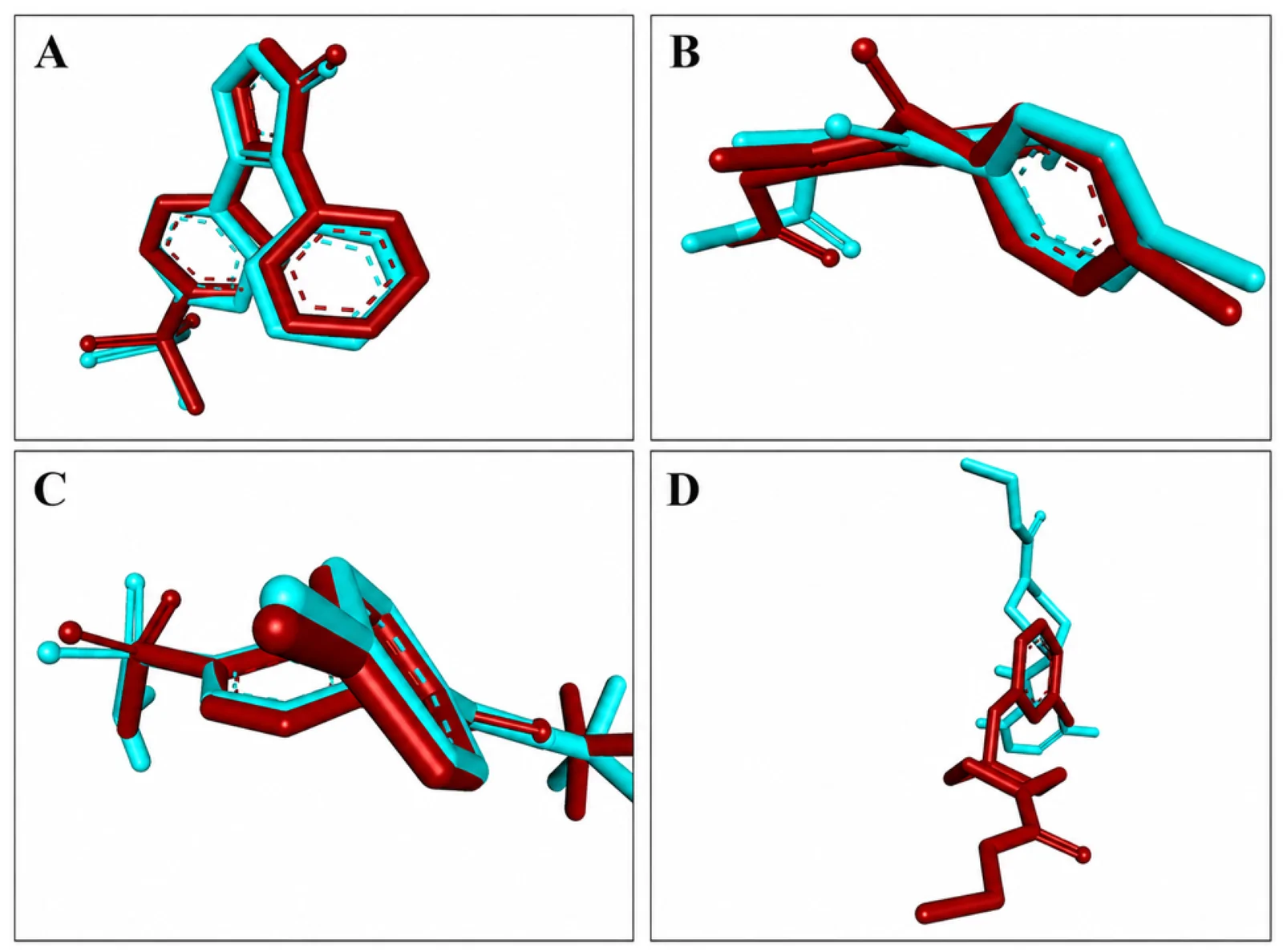
Superimposition of co-crystallized and re-docked ligand poses validating the docking protocol. The co-crystallized experimental ligand poses are represented as cyan sticks, while the re-docked predicted poses are represented as dark-red sticks. Dashed circular lines within the molecular rings denote standard aromaticity (delocalized π-electron systems). (**A**) Co-crystallized and re-docked rofecoxib in 5KIR (RMSD = 0.840 Å). (**B**) Co-crystallized and re-docked indomethacin in 4COX (RMSD = 0.592 Å). (**C**) Co-crystallized and re-docked celecoxib in 3LN1 (RMSD = 0.391 Å). (**D**) Co-crystallized and re-docked AR-C95791 in 3E7G (RMSD = 0.451 Å).

Docking protocol validation (Figure 2) was performed by re-docking each co-crystallized ligand into its corresponding target structure and superimposing the re-docked pose onto the experimentally determined conformation. The re-docked poses of rofecoxib (5KIR), indomethacin (4COX), celecoxib (3LN1), and AR-C95791 (3E7G) closely reproduced the orientations of their respective co-crystallized ligands, with RMSD values of 0.840 Å, 0.592 Å, 0.391 Å, and 0.451 Å (Figure 2). As all values fell below the commonly accepted 2 Å threshold for acceptable pose reproduction, the docking protocol was considered validated and suitable for predicting the binding orientations of the test compounds in subsequent analyses.

#### 2.2.2. Multi-Target Binding Affinity Profiling

##### Virtual Screening and Interaction Profiling Against Human COX-2 (PDB ID: 5KIR)

Structure-based virtual screening of all sixty-three constituents against the rofecoxib-defined active site of human COX-2 returned binding affinities spanning −8.1 to −4.5 kcal·mol^−1^ (Table 2). The top-ranked compound, 7-isopropyl-4,10-dimethylenecyclodecene (CID 6428525, ΔG = −7.9 kcal·mol^−1^), was closely followed by (Z,Z,Z)-1,5,9,9-tetramethyl-1,4,7-cycloundecatriene (CID 5281520, −7.9 kcal·mol^−1^) and Cardenolide (CID 53957771, −7.9 kcal·mol^−1^). Importantly, six of the ten highest-scoring constituents belonged to the hydrocarbon sesquiterpene family, mirroring the known geometric and lipophilic complementarity between such C_15_ scaffolds and the deep, predominantly hydrophobic COX-2 selectivity channel. The major oxygenated monoterpenes citronellal, citronellol, and isopulegol displayed moderate but reproducible affinities in the range of −6.0 to −6.6 kcal·mol^−1^; their high relative abundance (collectively exceeding 62% of the oil) suggests that they likely contribute additively or synergistically to the macroscopic anti-inflammatory activity of the unfractionated oil. Re-docking of the co-crystallized rofecoxib reproduced the experimental binding pose, confirming protocol validity.

**Figure 3:**
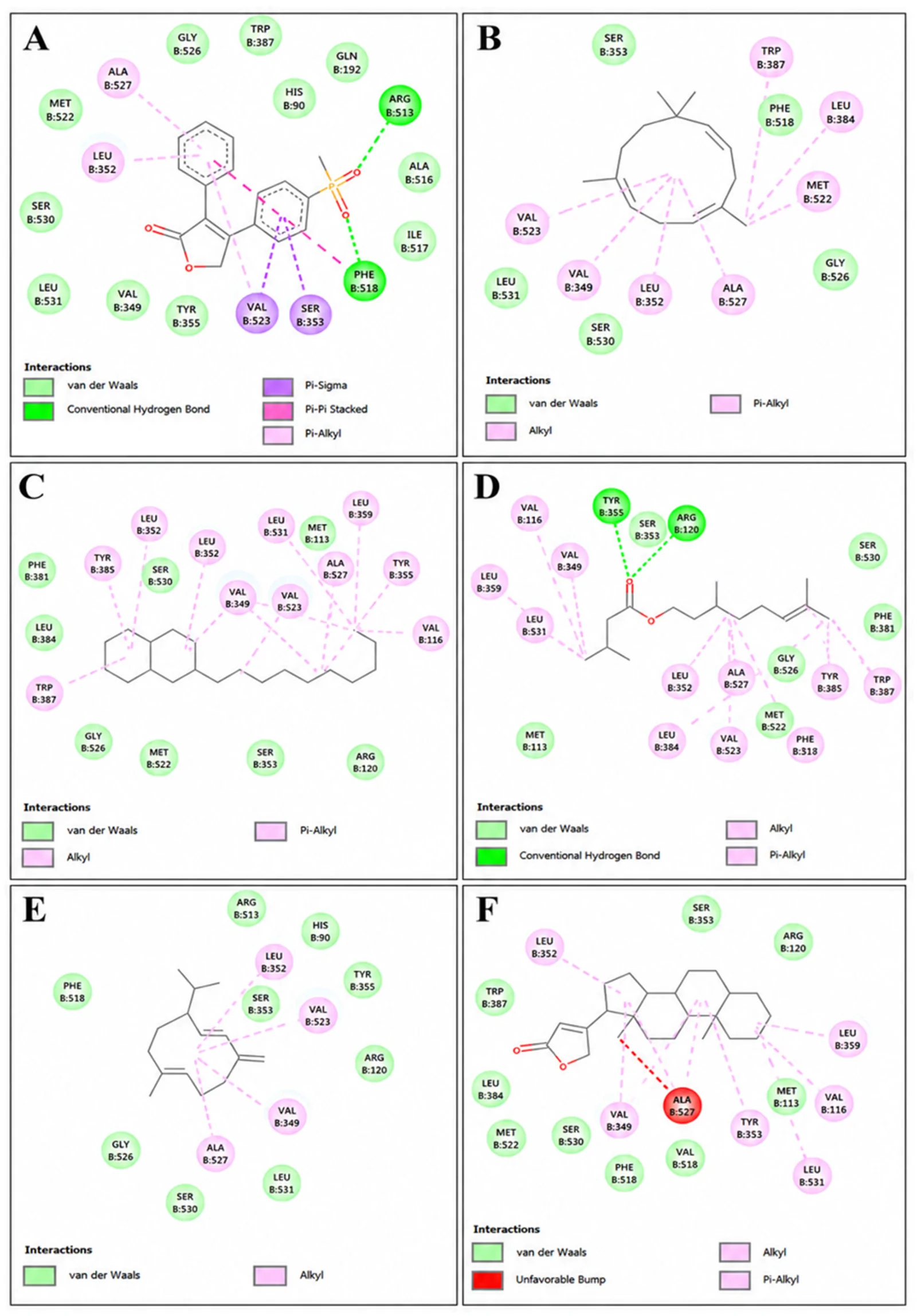
Predicted binding interactions of the co-crystallized ligand and screened test compounds docked into the 5KIR active site, illustrated as two-dimensional interaction maps. (**A**) Rofecoxib, the co-crystallized reference ligand; (**B**) (Z,Z,Z)-1,5,9,9-tetramethyl-1,4,7-cycloundecatriene; (**C**) 2-decyldecahydronaphthalene; (**D**) citronellyl isovalerate; (**E**) germacrene D; (**F**) cardenolide. In each panel, active-site residues are represented as colored discs and dashed lines denote the individual ligand–residue interactions, color-coded according to the interaction types listed in the corresponding panel legend; residues shown without connecting lines are involved in van der Waals contacts. Ligand atoms are colored by element following the standard convention (carbon in gray, oxygen in red, sulfur in yellow). Alkyl and π-alkyl interactions, both rendered in light pink by BIOVIA Discovery Studio Visualizer, correspond to hydrophobic contacts established between two aliphatic groups (alkyl) or between an aromatic π-system and an aliphatic group (π-alkyl), respectively. In panel (**F**), the red dashed line indicates an unfavorable steric bump with ALA B:527, which represents a destabilizing rather than a stabilizing contact. Interaction types differ between panels and are therefore reported in each individual legend.

Molecular docking analysis was performed to evaluate the binding potential of the selected compounds toward the 5KIR target protein (Table 3 and Figure 3), using the co-crystallized Rofecoxib (RCX) as the reference ligand. RCX exhibited the strongest binding affinity, with a docking score of −10.2 kcal/mol, and interacted with a broad network of residues, including HIS B:90, GLN B:192, VAL B:349, LEU B:352, SER B:353, TYR B:355, TRP B:387, ARG B:513, ALA B:516, ILE B:517, PHE B:518, MET B:522, VAL B:523, GLY B:526, ALA B:527, SER B:530, and LEU B:531.

Among the screened compounds, (Z,Z,Z)-1,5,9,9-tetramethyl-1,4,7-cycloundecatriene demonstrated the most favorable binding affinity (−8.0 kcal/mol), followed by 2-decyldecahydronaphthalene (−7.6 kcal/mol). Citronellyl isovalerate and Germacrene D exhibited comparable binding affinities of −7.2 kcal/mol, whereas Cardenolide showed substantially weaker binding (−2.8 kcal/mol). Thus, the first three candidates displayed considerably more favorable docking scores than Cardenolide.

Comparison of the interaction profiles with the co-crystallized RCX revealed substantial conservation of the binding-site residues. In particular, VAL B:349, LEU B:352, SER B:353, TYR B:355, TRP B:387, PHE B:518, MET B:522, VAL B:523, GLY B:526, ALA B:527, SER B:530, and LEU B:531 were repeatedly observed among the candidate–protein interactions and the reference complex. These conserved residues may contribute importantly to ligand recognition and stabilization within the 5KIR binding pocket.

Notably, (Z,Z,Z)-1,5,9,9-tetramethyl-1,4,7-cycloundecatriene combined the best docking score among the candidate compounds (−8.0 kcal/mol) with an interaction pattern highly overlapping with the reference ligand. 2-Decyldecahydronaphthalene also showed a favorable docking score and extensive conservation of the RCX-associated residues. Overall, the docking results identify (Z,Z,Z)-1,5,9,9-tetramethyl-1,4,7-cycloundecatriene and 2-decyldecahydronaphthalene as the most promising candidates, warranting further evaluation through molecular dynamics simulations and subsequent binding-energy analysis.

##### COX-2 (PDB: 3LN1)

**Figure 4:**
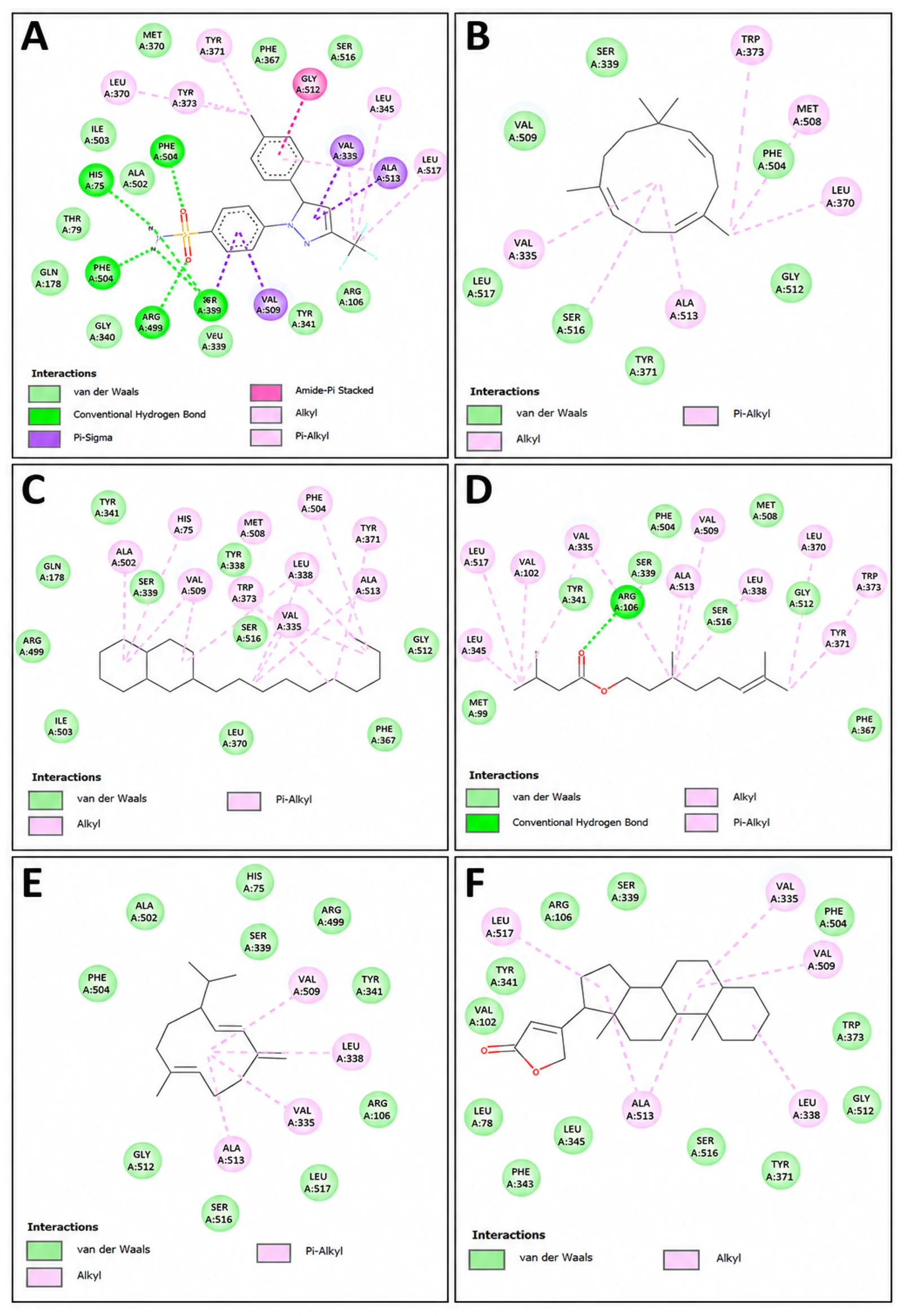
Two-dimensional protein–ligand interaction diagrams depicting the binding modes of the co-crystallized reference ligand and top-ranked test compounds within the active site of 3LN1. (**A**) Celecoxib, the co-crystallized reference ligand; (**B**) (Z,Z,Z)-1,5,9,9-tetramethyl-1,4,7-cycloundecatriene; (**C**) 2-decyldecahydronaphthalene; (**D**) citronellyl isovalerate; (**E**) germacrene D; (**F**) cardenolide. Residue discs, dashed interaction lines, and the alkyl/π-alkyl color convention are as defined in Figure 3. In panel (**A**), the nitrogen atoms of the pyrazole and sulfonamide groups are shown in blue and the fluorine atoms of the trifluoromethyl group in light blue. Interaction types differ between panels and are reported in each individual legend.

Molecular docking analysis (Table 4 and Figure 4) revealed that the co-crystallized ligand 3LN1–Celecoxib exhibited the strongest binding affinity (−12.2 kcal/mol) and interacted with 25 residues within the binding pocket. Among the investigated ligands, (Z,Z,Z)-1,5,9,9-tetramethyl-1,4,7-cycloundecatriene showed the most favorable predicted binding affinity (−8.2 kcal/mol), followed by 2-decyldecahydronaphthalene (−7.8 kcal/mol), Germacrene D (−7.7 kcal/mol), Citronellyl isovalerate (−7.2 kcal/mol), and Cardenolide (−6.4 kcal/mol). Comparison of their interacting residues with those of the co-crystallized ligand demonstrated substantial conservation of the binding-site interactions. Notably, 2-decyldecahydronaphthalene shared the highest number of common residues (18 residues; 72%) with the co-crystal interaction profile, followed by Citronellyl isovalerate (17 residues; 68%), Cardenolide (15 residues; 60%), Germacrene D (14 residues; 56%), and (Z,Z,Z)-1,5,9,9-tetramethyl-1,4,7-cycloundecatriene (13 residues; 52%). Several residues, including SER A:339, VAL A:335, LEU A:338, TYR A:341, PHE A:504, VAL A:509, GLY A:512, ALA A:513, SER A:516, and TRP A:373, were repeatedly observed across multiple ligand–protein complexes, suggesting their potential importance in stabilizing ligand binding within the 3NL1 binding pocket. Overall, -(Z,Z,Z)-1,5,9,9-Tetramethyl-1,4,7-cycloundecatriene demonstrated the most favorable combination of binding affinity and similarity to the co-crystallized ligand’s interaction pattern, indicating that it may adopt a binding mode closely resembling that of Celecoxib and warrant further investigation.

##### COX-1 (PDB: 4COX)

**Figure 5:**
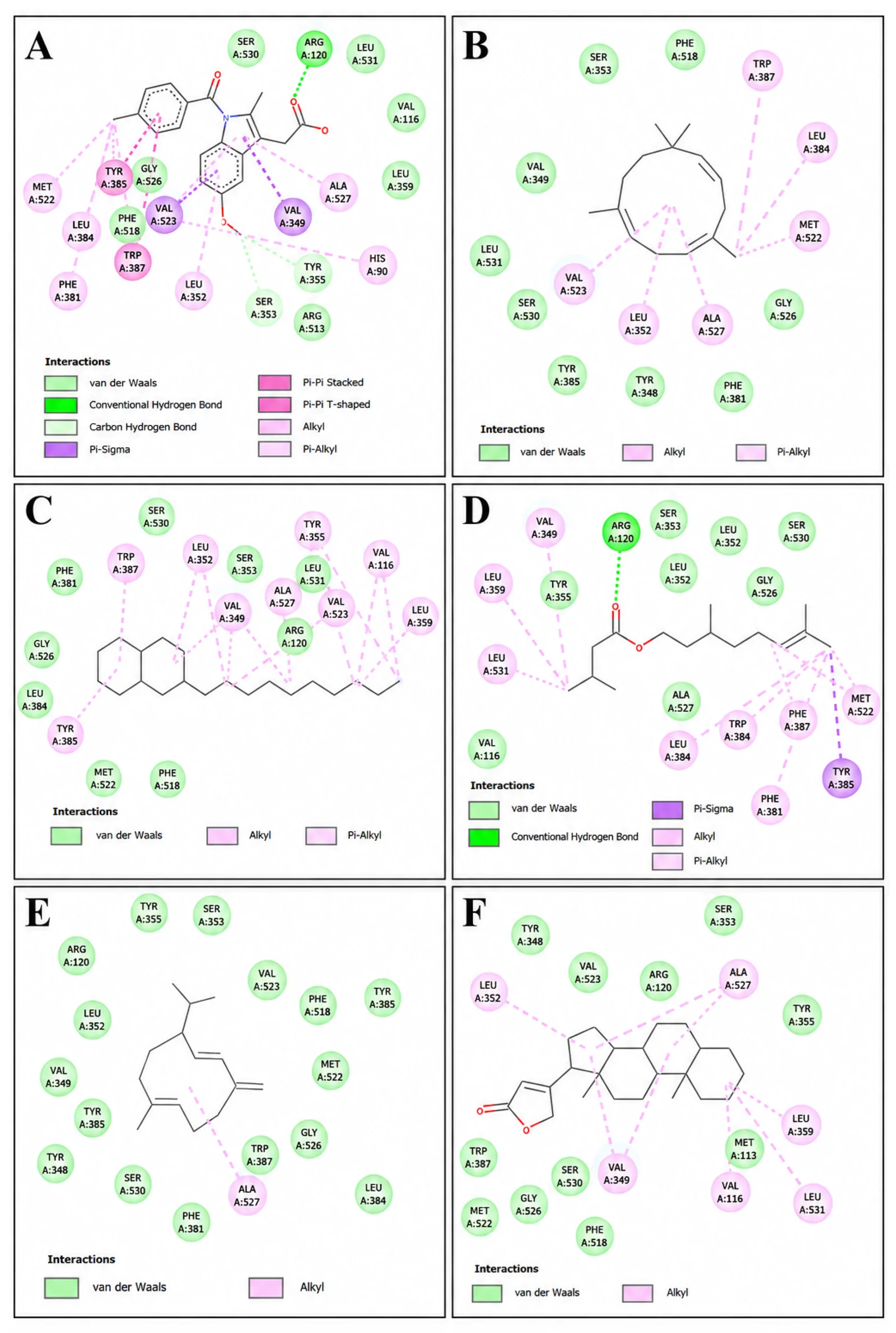
Two-dimensional depiction of ligand–residue interactions within the 4COX binding pocket for the co-crystallized reference and the top-ranked test compounds. (**A**) Indomethacin, the native co-crystallized ligand; (**B**) (Z,Z,Z)-1,5,9,9-tetramethyl-1,4,7-cycloundecatriene; (**C**) 2-decyldecahydronaphthalene; (**D**) citronellyl isovalerate; (**E**) germacrene D; (**F**) cardenolide. Residue discs, dashed interaction lines, and the alkyl/π-alkyl color convention are as defined in Figure 3; the indole nitrogen is shown in blue. π–π stacked and π–π T-shaped interactions are likewise rendered in identical colors, and denote aromatic contacts adopting a parallel or a perpendicular edge-to-face orientation, respectively. Interaction types differ between panels and are reported in each individual legend.

The docking analysis (Table 5 and Figure 5) showed that the 4COX–indomethacin co-crystal complex had the most favorable binding affinity of −9.6 kcal/mol, establishing the reference interaction profile. Among the screened ligands, (Z,Z,Z)-1,5,9,9-tetramethyl-1,4,7-cycloundecatriene showed the strongest predicted binding (−8.8 kcal/mol), followed by 2-decyldecahydronaphthalene (−8.5 kcal/mol) and Germacrene D (−8.3 kcal/mol). Citronellyl isovalerate showed a binding affinity of −7.6 kcal/mol, whereas Cardenolide exhibited the weakest binding (−6.7 kcal/mol).

Importantly, the top-ranked ligands reproduced a substantial portion of the co-crystal binding-site residues, suggesting that they occupy a similar region of the 4COX binding pocket. The 2-decyldecahydronaphthalene and Citronellyl isovalerate complexes shared 18 residues with the co-crystal complex, while (Z,Z,Z)-1,5,9,9-tetramethyl-1,4,7-cycloundecatriene shared 15 residues. Germacrene D shared 14 residues, despite its relatively favorable binding affinity of −8.3 kcal/mol. Cardenolide also reproduced 18 co-crystal residues, although its overall binding affinity was considerably weaker.

Across the ligand complexes, residues such as SER A:353, VAL A:349, LEU A:352, TYR A:355, TRP A:387, PHE A:518, MET A:522, VAL A:523, GLY A:526, ALA A:527, SER A:530, and LEU A:531 appeared repeatedly, indicating that these residues may represent important components of the 4COX ligand-binding pocket. Overall, the combination of favorable binding affinity and extensive overlap with the co-crystal interaction profile particularly supports (Z,Z,Z)-1,5,9,9-tetramethyl-1,4,7-cycloundecatriene, 2-decyldecahydronaphthalene, and Germacrene D as promising 4COX-binding candidates for subsequent structural and molecular-dynamics analysis.

##### Human iNOS (PDB: 3E7G)

**Figure 6:**
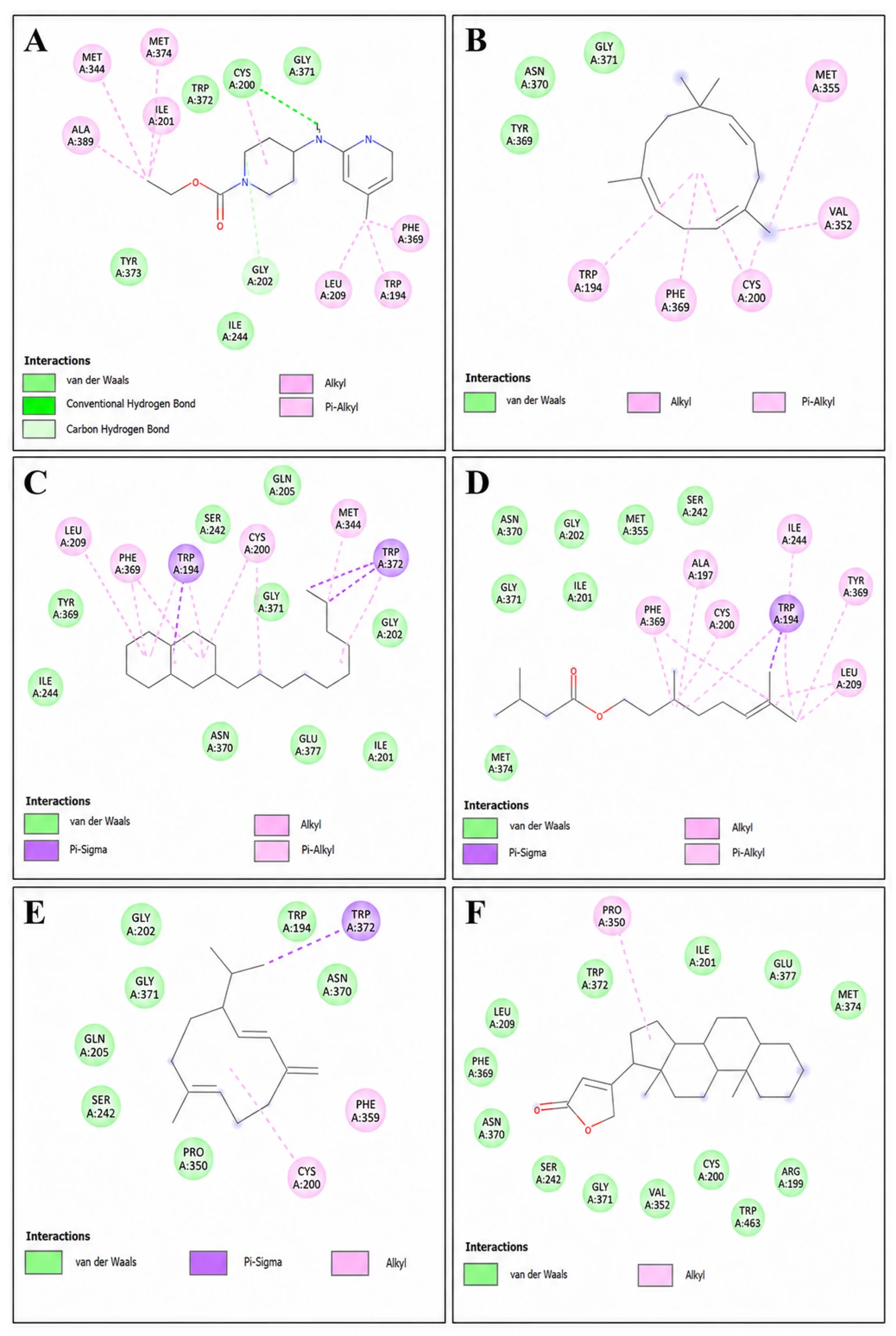
Two-dimensional interaction diagrams comparing the binding profile of the co-crystallized ligand with that of the docked test compounds within the 3E7G active site. (**A**) AR-C95791, the co-crystallized reference ligand; (**B**) (Z,Z,Z)-1,5,9,9-tetramethyl-1,4,7-cycloundecatriene; (**C**) 2-decyldecahydronaphthalene; (**D**) citronellyl isovalerate; (**E**) germacrene D; (**F**) cardenolide. Residue discs, dashed interaction lines, and the alkyl/π-alkyl color convention are as defined in Figure 3; the nitrogen atoms of the piperidine ring, the amine linker and the pyridine ring are shown in blue. Interaction types differ between panels and are reported in each individual legend.

The molecular docking analysis of 3E7G (Table 6 and Figure 6) with the selected ligands demonstrated favorable interactions within the binding pocket. The co-crystal ligand AR-C95791 exhibited a binding affinity of −8.4 kcal/mol and interacted with key residues including CYS200, ILE201, TRP194, PHE369, GLY371, TRP372, MET374, MET434, TYR489, and LEU209. Among the screened ligands, Cardenolide showed the strongest predicted binding affinity (−8.8 kcal/mol), followed by (Z,Z,Z)-1,5,9,9-tetramethyl-1,4,7-cycloundecatriene (−6.8 kcal/mol), 2-decyldecahydronaphthalene (−8.2 kcal/mol), citronellyl isovalerate (−6.9 kcal/mol), and germacrene D (−7.2 kcal/mol). Notably, 2-decyldecahydronaphthalene shared 12 residues with the co-crystal ligand, representing the highest interaction overlap, whereas citronellyl isovalerate and Cardenolide shared 9 and 8 residues, respectively. The residues CYS200, GLY371, PHE369, and TRP194 were consistently involved in interactions with multiple ligands, suggesting their importance in ligand recognition within the binding pocket. Overall, Cardenolide demonstrated the most favorable docking affinity, while 2-decyldecahydronaphthalene exhibited the greatest similarity to the co-crystal interaction pattern.

### 2.3. Comparative Quantum Chemical Profiling of Top Candidates

**Figure 7:**
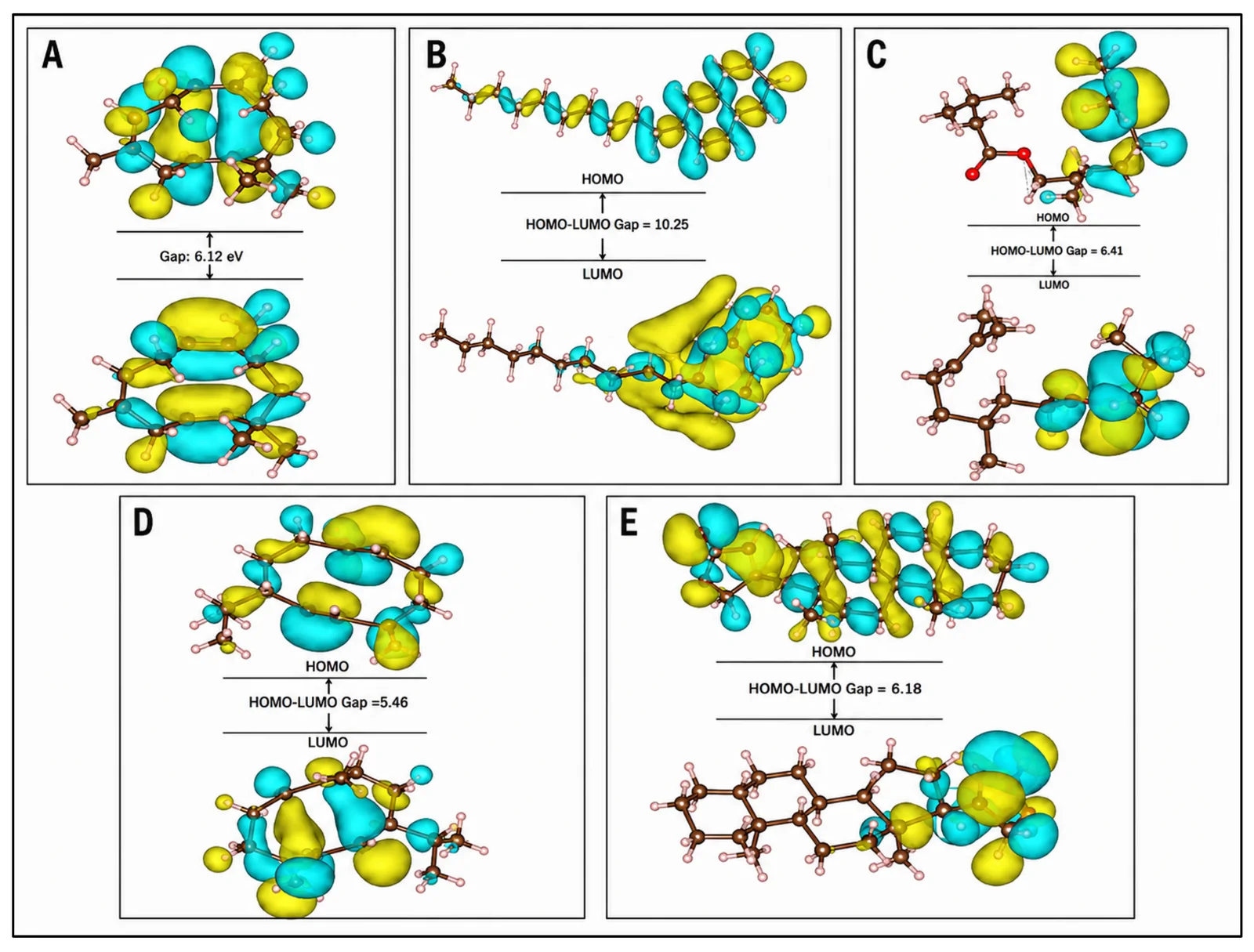
Frontier molecular orbital (FMO) analysis showing the spatial distribution of the Highest Occupied Molecular Orbital (HOMO) and Lowest Unoccupied Molecular Orbital (LUMO) surfaces calculated at the B3LYP/3-21G level for the top five candidate compounds. The cyan and yellow isosurfaces represent the positive and negative phases of the molecular orbital wavefunctions, respectively. In the energy-level diagrams, solid horizontal lines represent the discrete molecular energy levels, and vertical arrows denote the calculated energy gaps (ΔE). (**A**) (Z,Z,Z)-1,5,9,9-tetramethyl-1,4,7-cycloundecatriene, (**B**) 2-decyldecahydronaphthalene, (**C**) citronellyl isovalerate, (**D**) germacrene D, and (**E**) cardenolide. The calculated HOMO–LUMO energy gaps (ΔE) reflect the relative electronic stability, chemical hardness, and reactivity profiles of each molecular scaffold.

Unconstrained geometry optimizations of the top five candidate compounds (Z,Z,Z)-1,5,9,9-tetramethyl-1,4,7-cycloundecatriene,2-decyldecahydronaphthalene, citronellyl isovalerate, germacrene D, and cardenolide at the B3LYP/3-21G level within a CPCM solvation framework converged to true potential energy minima, as confirmed by the complete absence of imaginary vibrational frequencies. Analysis of the frontier molecular orbitals (FMOs) revealed marked electronic differences among the candidates, reflecting their distinct chemical structures (Figure 7 and Table 7).

The HOMO–LUMO energy gap (ΔE), a fundamental indicator of kinetic stability and chemical softness, spanned from 5.46 eV to 10.25 eV across the series. 2-Decyldecahydronaphthalene exhibited the largest energy gap (ΔE=10.25 eV), consistent with its fully saturated, non-conjugated bicyclic framework and high kinetic stability. Conversely, germacrene D displayed the narrowest gap (ΔE=5.46 eV) and highest HOMO energy ($E_{\mathrm{HOMO}}=-5.48\;\mathrm{eV}$), indicating greater electronic polarizability and a higher propensity for electron donation due to its delocalized triene framework. The lead sesquiterpene, (Z,Z,Z)-1,5,9,9-tetramethyl-1,4,7-cycloundecatriene, presented a substantial gap ($\Delta E=6.12\;\mathrm{eV}{,E}_{\mathrm{HOMO}}=-5.62\;\mathrm{eV}$, $E_{\mathrm{LUMO}}=0.50\;\mathrm{eV}$), with the FMO density localized predominantly over its endocyclic C=C double bonds. This electronic topology indicates that any photoredox or chemical reactivity is strictly confined to the olefinic core, leaving the saturated hydrocarbon boundary chemically inert.

Derived global reactivity descriptors further illuminated the chemical behavior of the series (Table 7). Chemical hardness (η) and softness (S) confirmed 2-decyldecahydronaphthalene as the hardest, least polarizable scaffold (η=5.13 eV), while germacrene D emerged as the softest (η=2.73 eV). Cardenolide possessed a positive electron affinity (A=0.90 eV), leading to the highest electronegaivity (χ=3.99 eV) and global electrophilicity index (ω=2.58 eV) among all candidates, alongside a large dipole moment (7.17 Debye) driven by its oxygenated lactone ring. In contrast, the lead sesquiterpene (Z,Z,Z)-1,5,9,9-tetramethyl-1,4,7-cycloundecatriene displayed a hard, weakly polarizable profile (η=3.06 eV, $S=0.163{\;\mathrm{eV}}^{-1}$, ω=1.07 eV) with a small dipole moment (0.6929 Debye), indicating a symmetric, non-polar charge distribution.

**Figure 8:**
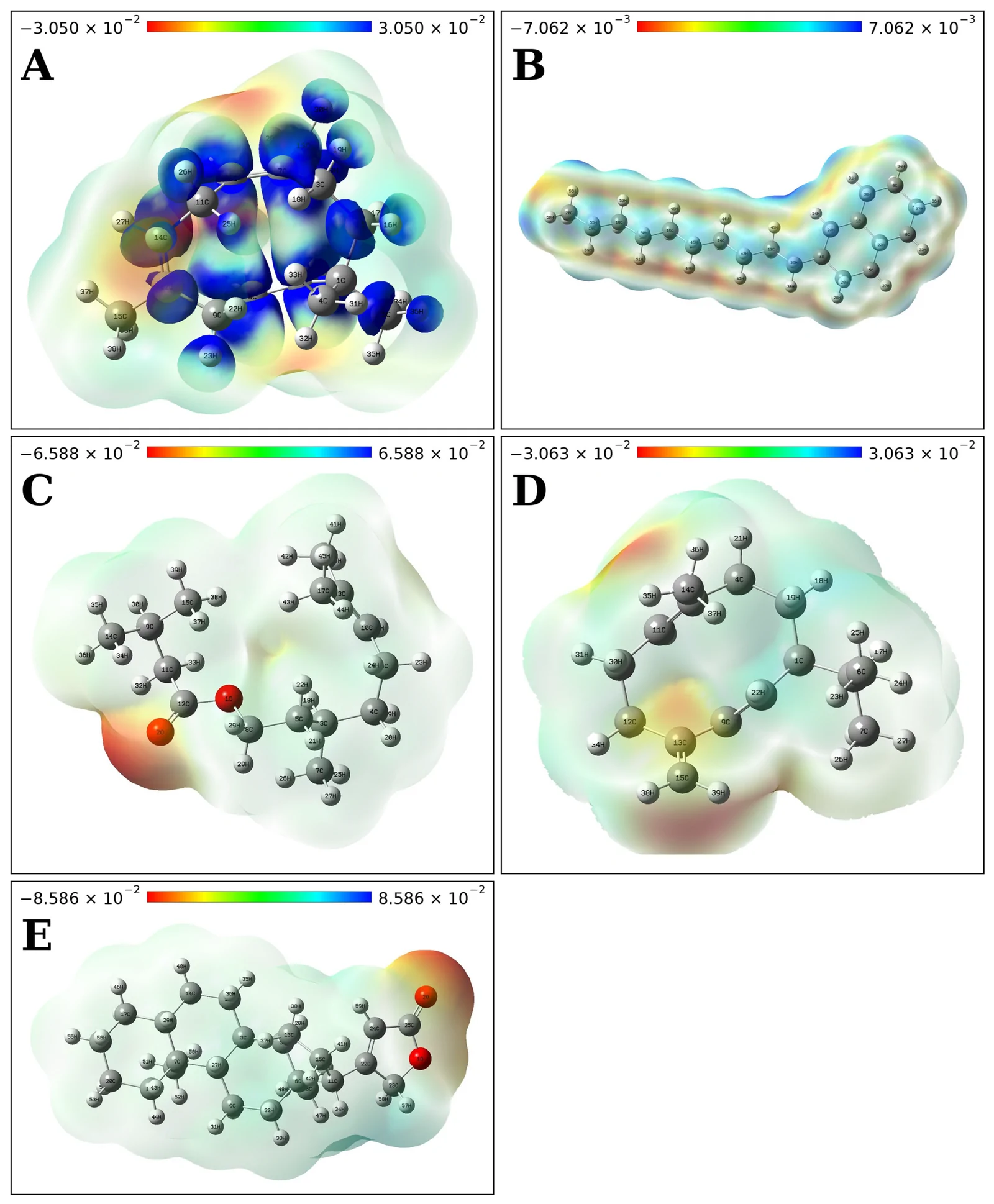
Molecular electrostatic potential (MEP) surfaces of the top candidate compounds mapped onto their total electron density at the B3LYP/3-21G/CPCM level: (**A**) (Z,Z,Z)-1,5,9,9-tetramethyl-1,4,7-cycloundecatriene, (**B**) 2-decyldecahydronaphthalene, (**C**) citronellyl isovalerate, (**D**) germacrene D, and (**E**) cardenolide. The color scale spans from red (strongly negative/electron-rich regions representing nucleophilic sites and hydrogen-bond acceptors) to blue (strongly positive/electron-poor regions representing electrophilic sites and hydrogen-bond donors), with green denoting neutral electrostatic potential.

Molecular Electrostatic Potential (MEP) mapping provided a spatial depiction of nucleophilic and electrophilic susceptibility across the molecular surfaces (Figure 8). Cardenolide exhibited intense negative electrostatic potential (red regions) over its lactone carbonyl oxygen, marking it as a primary hydrogen-bond acceptor, while positive potential (blue regions) concentrated around its hydroxyl protons. In contrast, the MEP maps for (Z,Z,Z)-1,5,9,9-tetramethyl-1,4,7-cycloundecatriene and 2-decyldecahydronaphthalene were dominated by neutral green regions, with only shallow, localized π-electron polarization over the olefinic double bonds. These quantum chemical characteristics establish that the lead sesquiterpene acts as a kinetically robust, non-covalent inhibitor whose binding within the COX active site is driven overwhelmingly by hydrophobic dispersion and shape complementarity rather than by polar or charge-transfer interactions.

### 2.4. Structural Stability of the Complex from MD Simulation

#### 2.4.1. RMSD and RMSF Analyses

**Figure 9:**
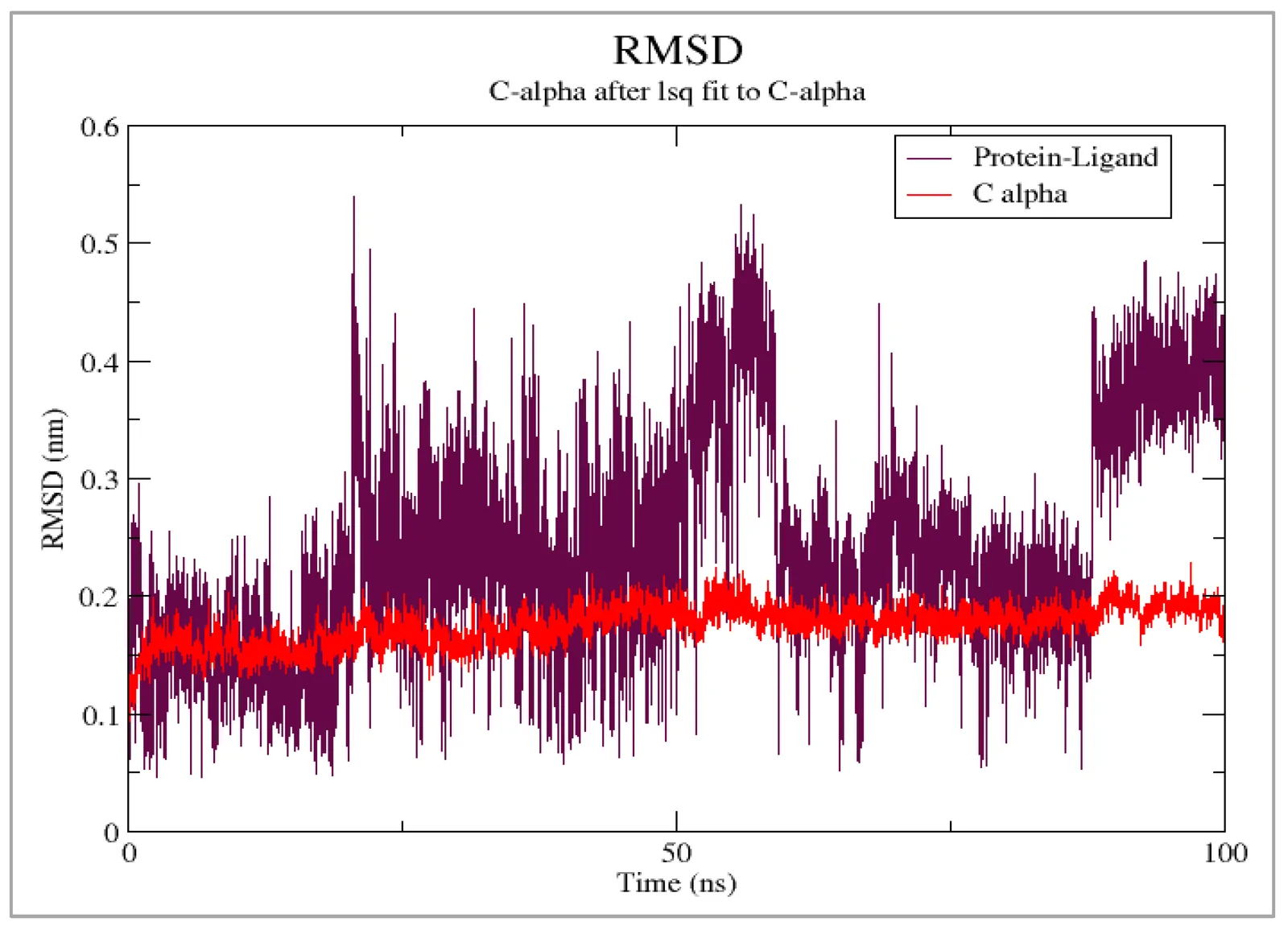
Time evolution of the root-mean-square deviation (RMSD) of the COX-2–ligand complex during the 100 ns molecular dynamics simulation. Mean Cα RMSD = 0.17 ± 0.02 nm; whole-complex RMSD = 0.24 ± 0.009 nm.

**Figure 10:**
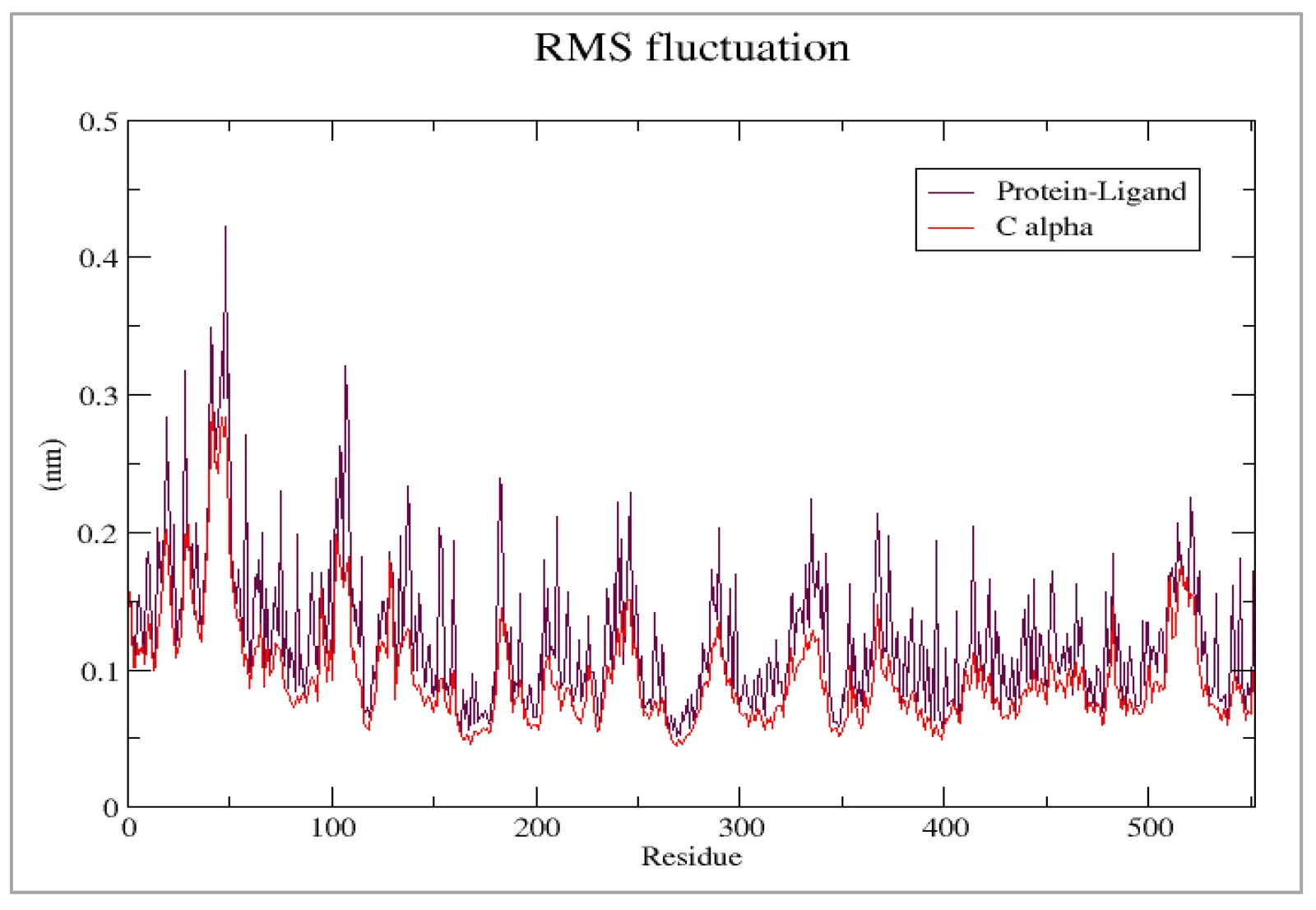
Per-residue root-mean-square fluctuation (RMSF) profile of the COX-2–ligand complex obtained from the production trajectory. Active-site residues exhibit reduced flexibility relative to surface loops.

The structural integrity of the COX-2–ligand complex was assessed throughout the 100 ns production trajectory. The Cα backbone of the protein equilibrated rapidly during the first 10 ns and subsequently fluctuated around a mean RMSD value of 0.17 ± 0.02 nm, while the entire complex displayed a slightly higher but equally well-converged average RMSD of 0.24 ± 0.009 nm (Figure 9). These low-amplitude deviations indicate that ligand binding did not induce any global structural reorganization of the enzyme. The residue-level flexibility profile (Figure 10) was likewise modest, with mean RMSF values of 0.09 ± 0.03 nm for the Cα atoms and 0.12 ± 0.05 nm for all atoms. Localized fluctuations were confined to peripheral loops distant from the active site, whereas the residues lining the binding pocket including those identified by docking remained rigid throughout the simulation, consistent with a stable enzyme–inhibitor recognition event.

#### 2.4.2. Radius of Gyration and Solvent-Accessible Surface Area

**Figure 11:**
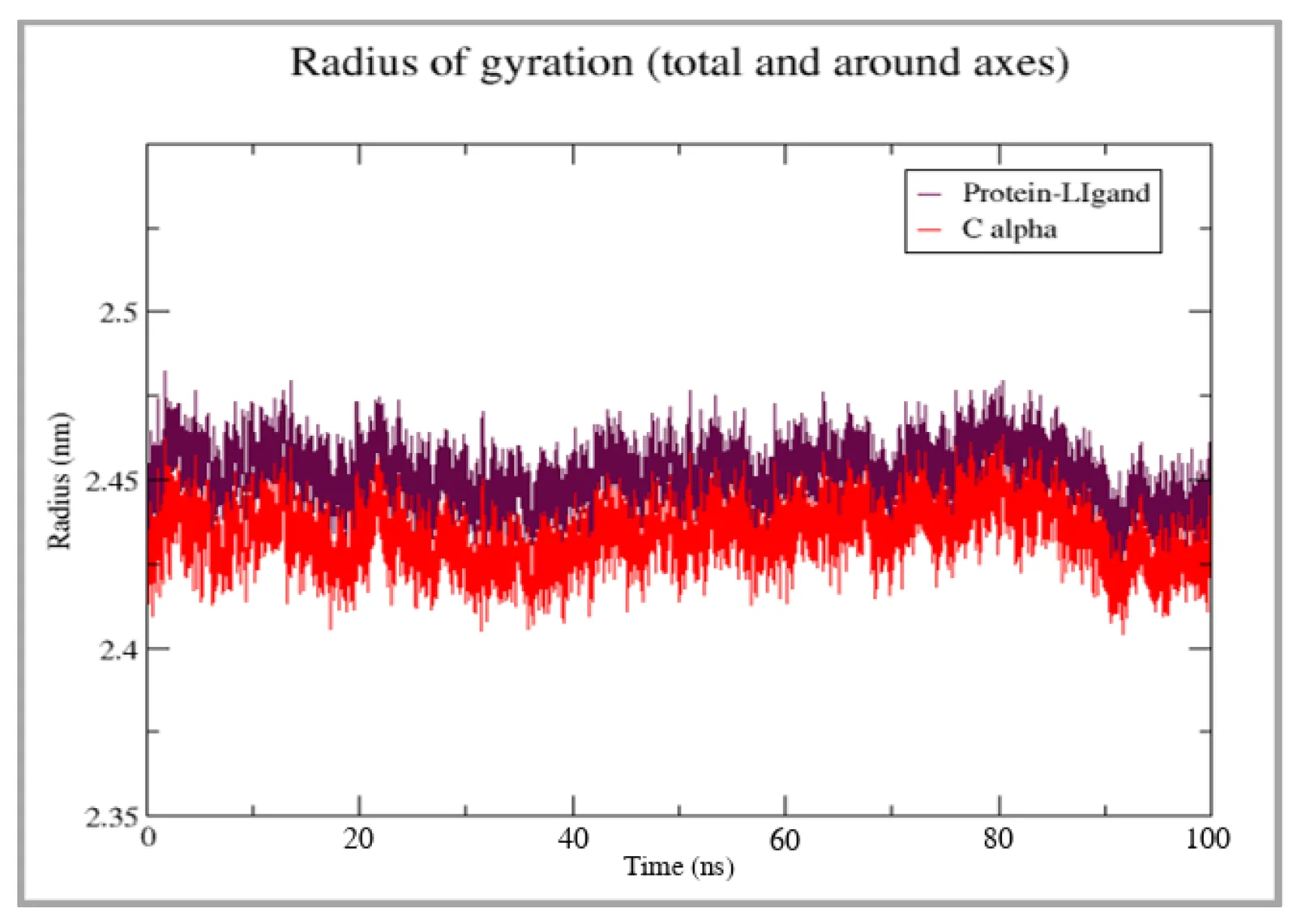
Radius of gyration (Rg) of the COX-2–ligand complex over the 100 ns trajectory. Both whole-complex (2.45 ± 0.008 nm) and Cα-backbone (2.43 ± 0.008 nm) values indicate sustained structural compactness.

**Figure 12:**
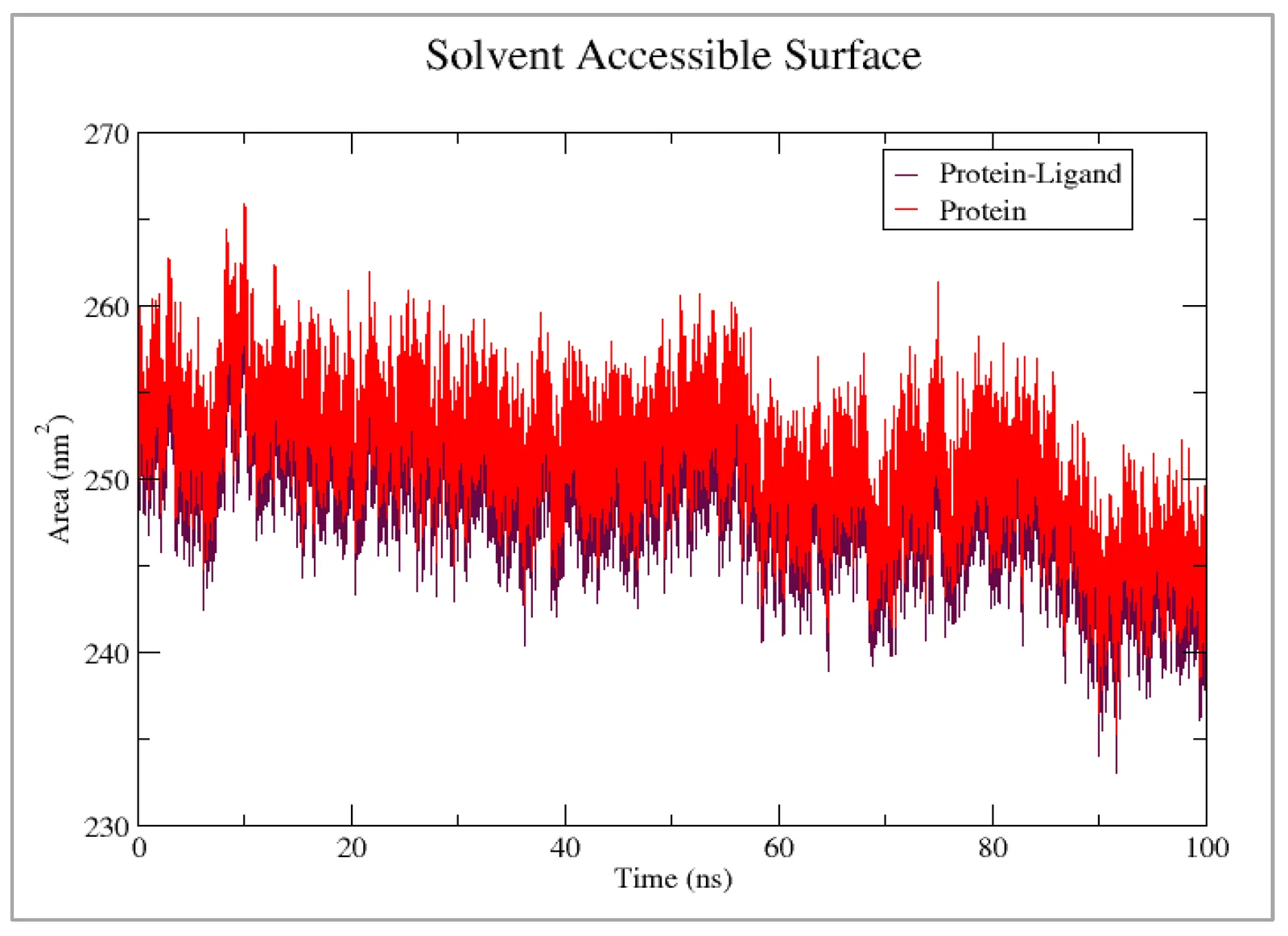
Solvent-accessible surface area (SASA) profiles of the apo COX-2 (251.11 ± 3.92 nm^2^) and the ligand-bound complex (248.90 ± 3.91 nm^2^), reflecting partial burial of binding-site residues upon ligand recognition.

The radius of gyration (Rg) provides a global descriptor of protein compactness. Both the Cα backbone (Rg = 2.43 ± 0.008 nm) and the whole complex (Rg = 2.45 ± 0.008 nm) maintained nearly invariant compactness throughout the simulation (Figure 11), with no detectable swelling, collapse, or domain rearrangement events. In a complementary fashion, the solvent-accessible surface area (SASA) of the ligand-bound complex (248.90 ± 3.91 nm^2^) was systematically lower than that of the apo enzyme (251.11 ± 3.92 nm^2^) (Figure 12), indicating a modest ligand-induced burial of pocket-lining residues consistent with stable accommodation within the cyclooxygenase channel. These structural observables collectively demonstrate that the binding of the sesquiterpene ligand stabilizes, rather than perturbs, the global fold of COX-2.

### 2.5. Essential Dynamics, Cross-Correlations, and Free Energy Landscape

**Figure 13:**
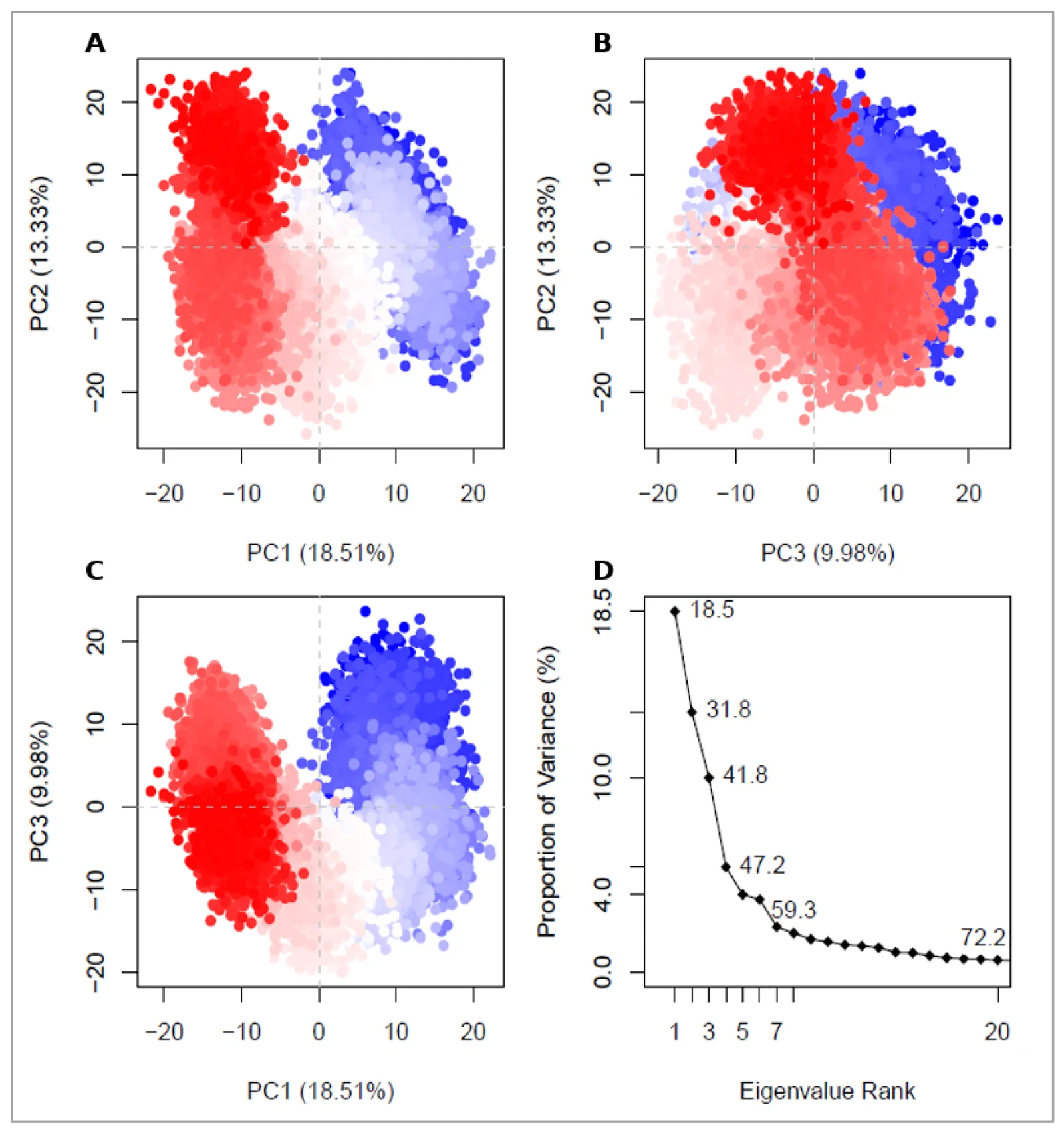
Principal component analysis (PCA) of the COX-2–ligand complex. The three scatter plots illustrate the conformational projections of the trajectory along (**A**) PC1 vs. PC2, (**B**) PC3 vs. PC2, and (**C**) PC1 vs. PC3, where the color gradient from red to blue (through white) represents the chronological time progression of the 100 ns simulation (from 0 ns in red to 100 ns in blue). (**D**) The bottom-right scree plot shows the proportion of variance explained by each eigenvalue rank, with the cumulative variance reaching 72.2% at the 20th rank.

**Figure 14:**
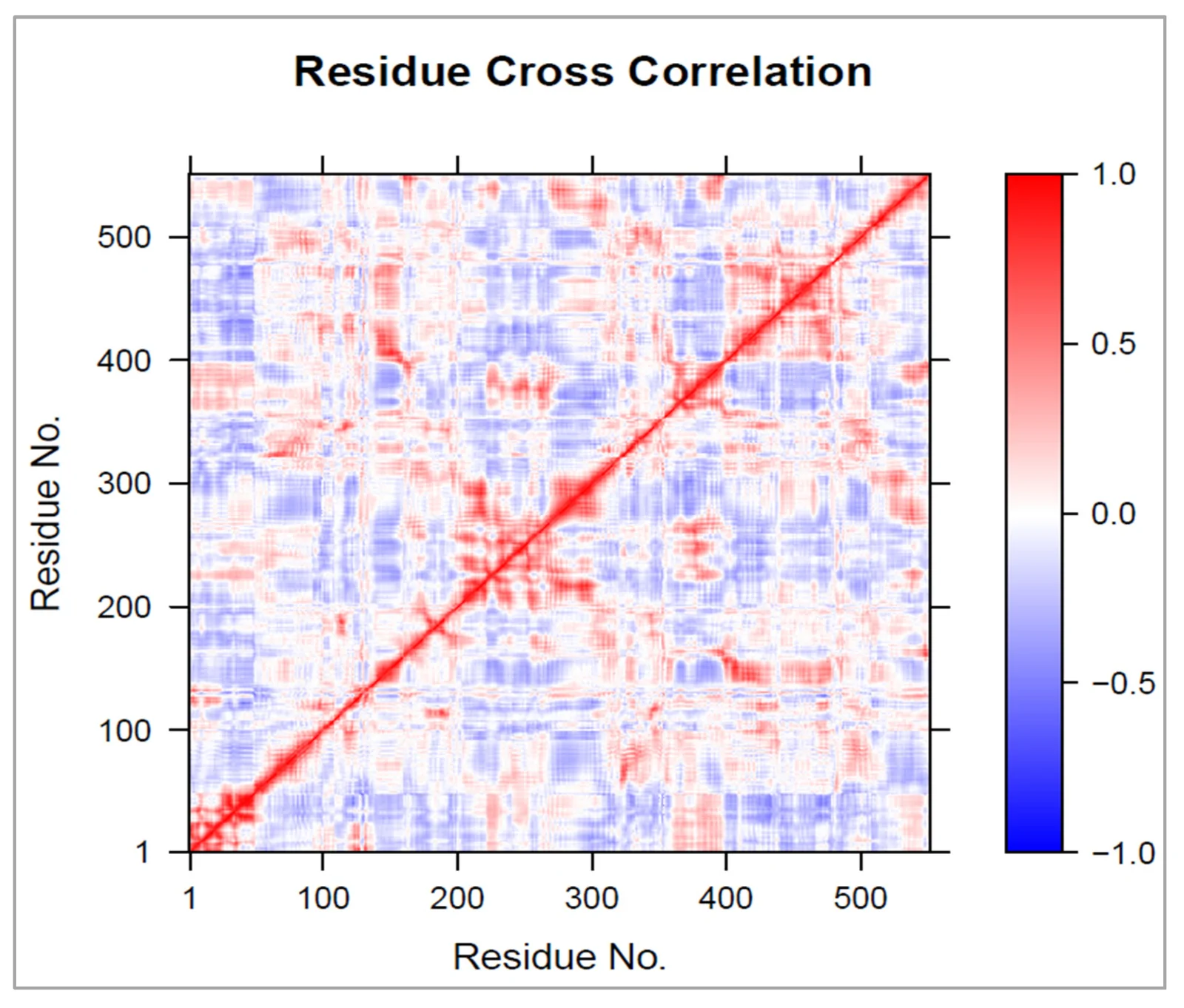
Dynamic cross-correlation matrix (DCCM) of Cα motions extracted from the production trajectory. The prominent solid red diagonal line represents the perfect self-correlation of each residue with itself ($C_{i,i}=1$). Off-diagonal positive (red) and negative (blue) regions correspond to correlated (in-phase) and anti-correlated (out-of-phase) residue motions, respectively.

**Figure 15:**
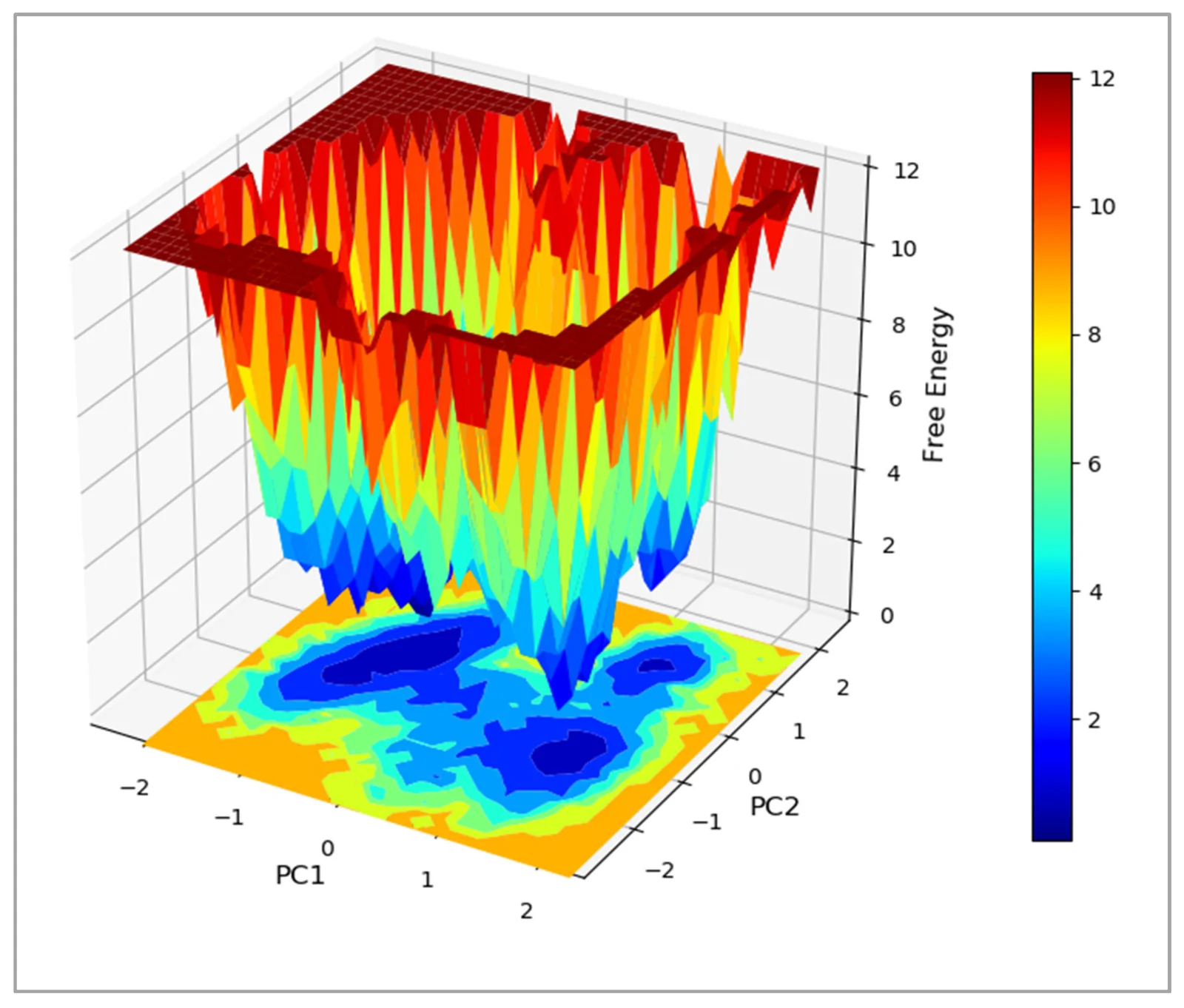
Gibbs free energy landscape of the COX-2–ligand complex projected onto the first two principal components (PC1, PC2). Three connected low-energy basins denote stable, energetically equivalent conformational states.

Principal component analysis (PCA) was employed to extract the dominant collective motions of the complex. The first three eigenvectors captured 18.51%, 13.33%, and 9.98% of the total variance, jointly describing 41.82% of the essential motion of the system (Figure 13). The two-dimensional projection of the trajectory onto the PC1–PC2 subspace revealed a tightly bounded conformational cloud, indicative of a single dominant attractor basin and the absence of large-scale rearrangements. Inspection of the dynamic cross-correlation matrix (DCCM, Figure 14) further confirmed this picture: regions of strong positive correlation, indicating coordinated motions of structurally adjacent secondary structure elements, dominated the matrix, while anti-correlated motions remained sparse and spatially restricted. The associated Gibbs free energy landscape, projected onto the first two principal components (Figure 15), exhibited three contiguous low-energy basins separated by shallow barriers. This topology is characteristic of a well-equilibrated complex that fluctuates between closely related, energetically equivalent conformers without undergoing functionally disruptive transitions.

### 2.6. End-Point Binding Free Energies and Per-Residue Decomposition

**Figure 16:**
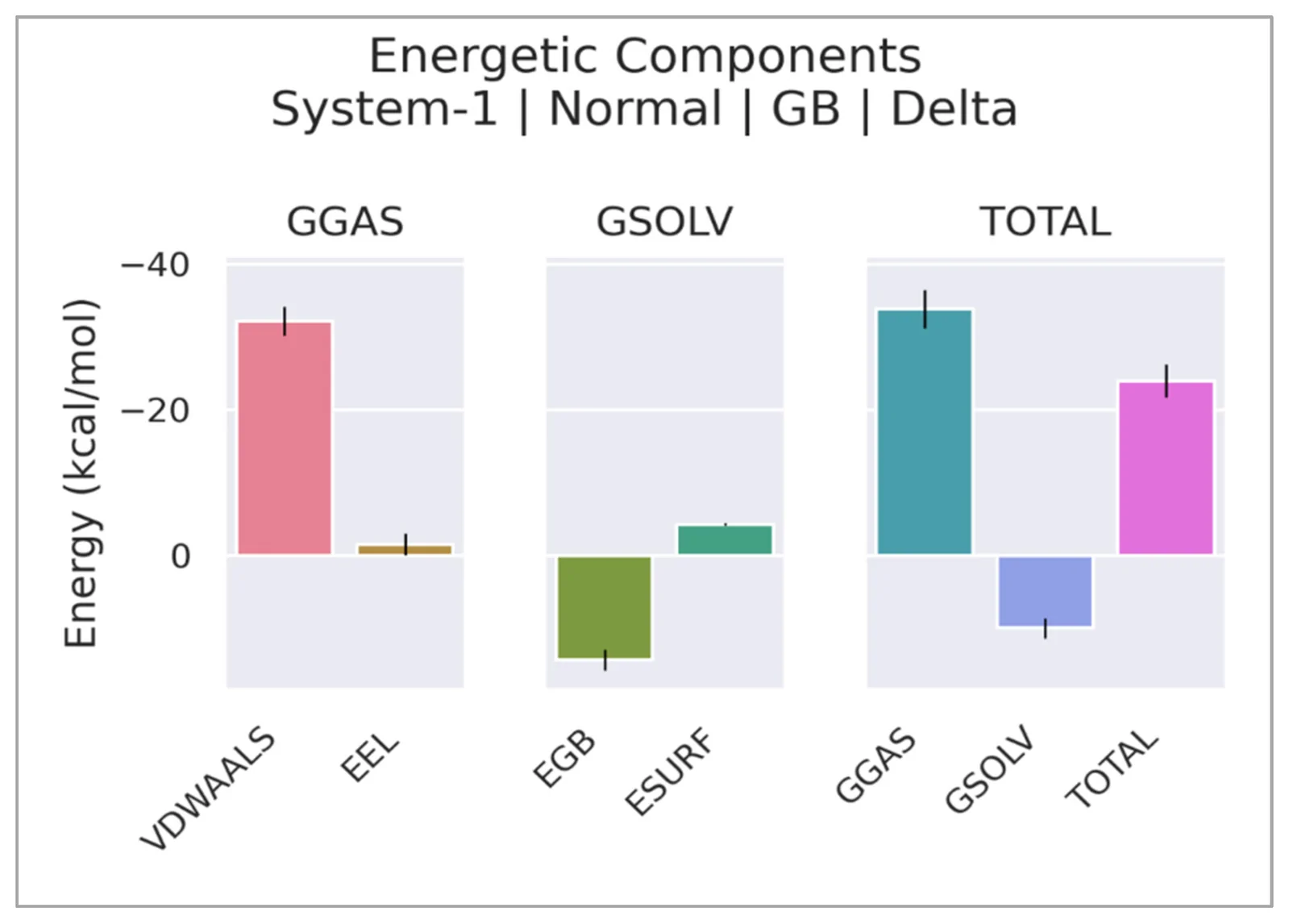
Total MM/GBSA binding free energy of the COX-2–ligand complex (ΔG_bind = −23.98 kcal·mol^−1^), partitioned into van der Waals, electrostatic, polar solvation, and non-polar solvation contributions.

**Figure 17:**
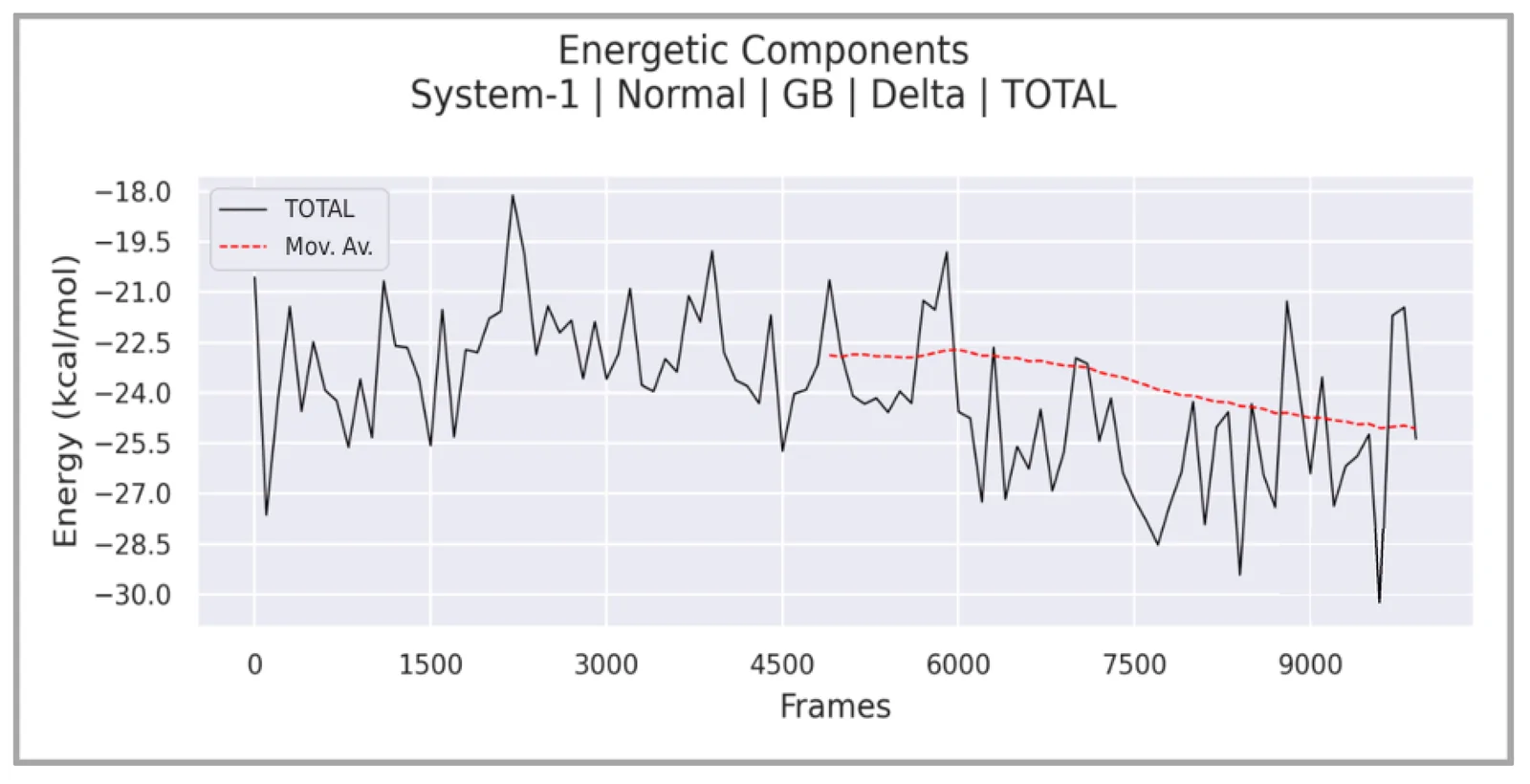
Frame-resolved MM/GBSA binding free energy along the production trajectory, illustrating the temporal stability of ligand binding.

**Figure 18:**
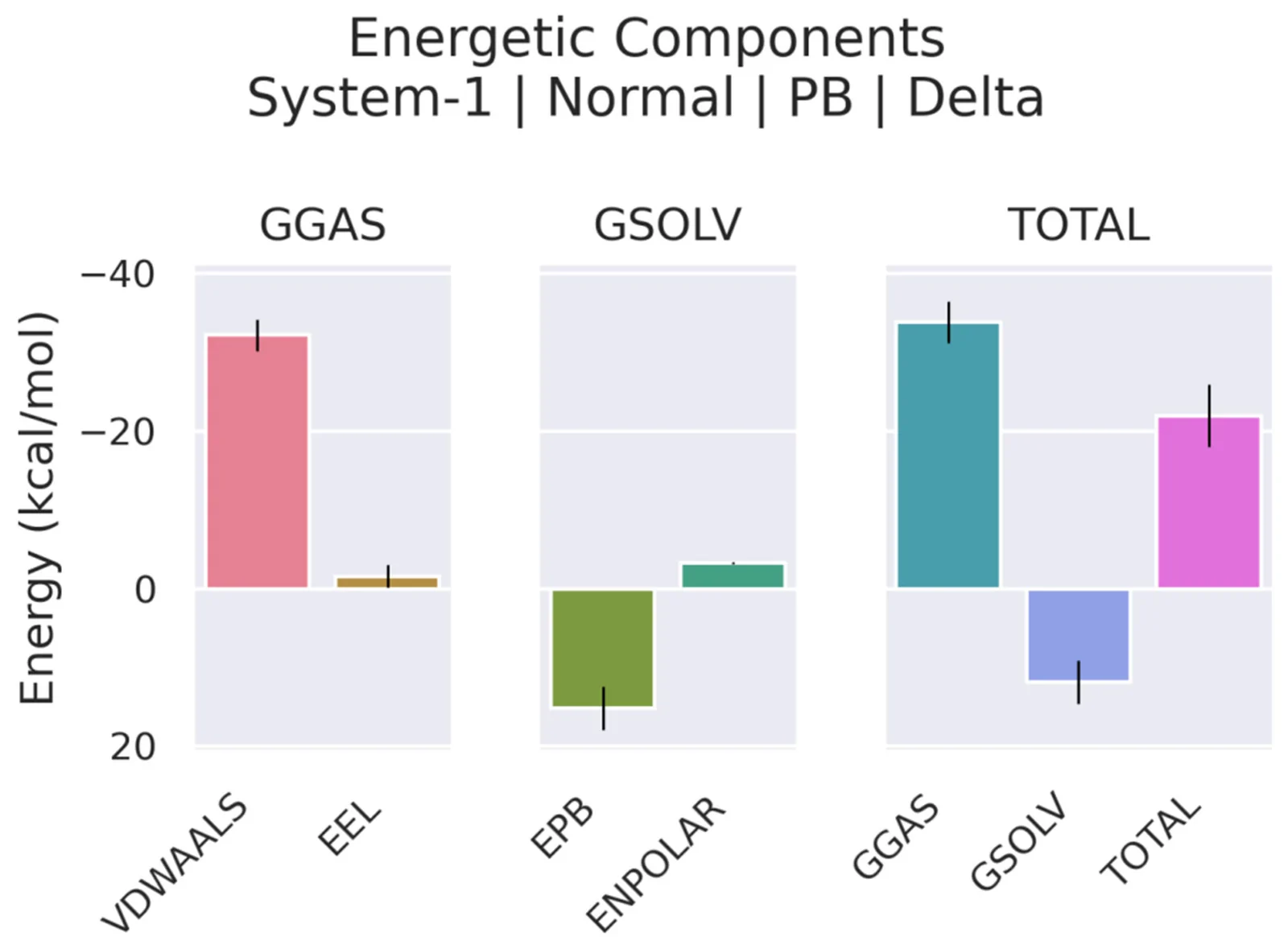
Total MM/PBSA binding free energy of the COX-2–ligand complex (ΔG_bind = −21.95 kcal·mol^−1^), partitioned into van der Waals, electrostatic, polar solvation, and non-polar solvation contributions.

**Figure 19:**
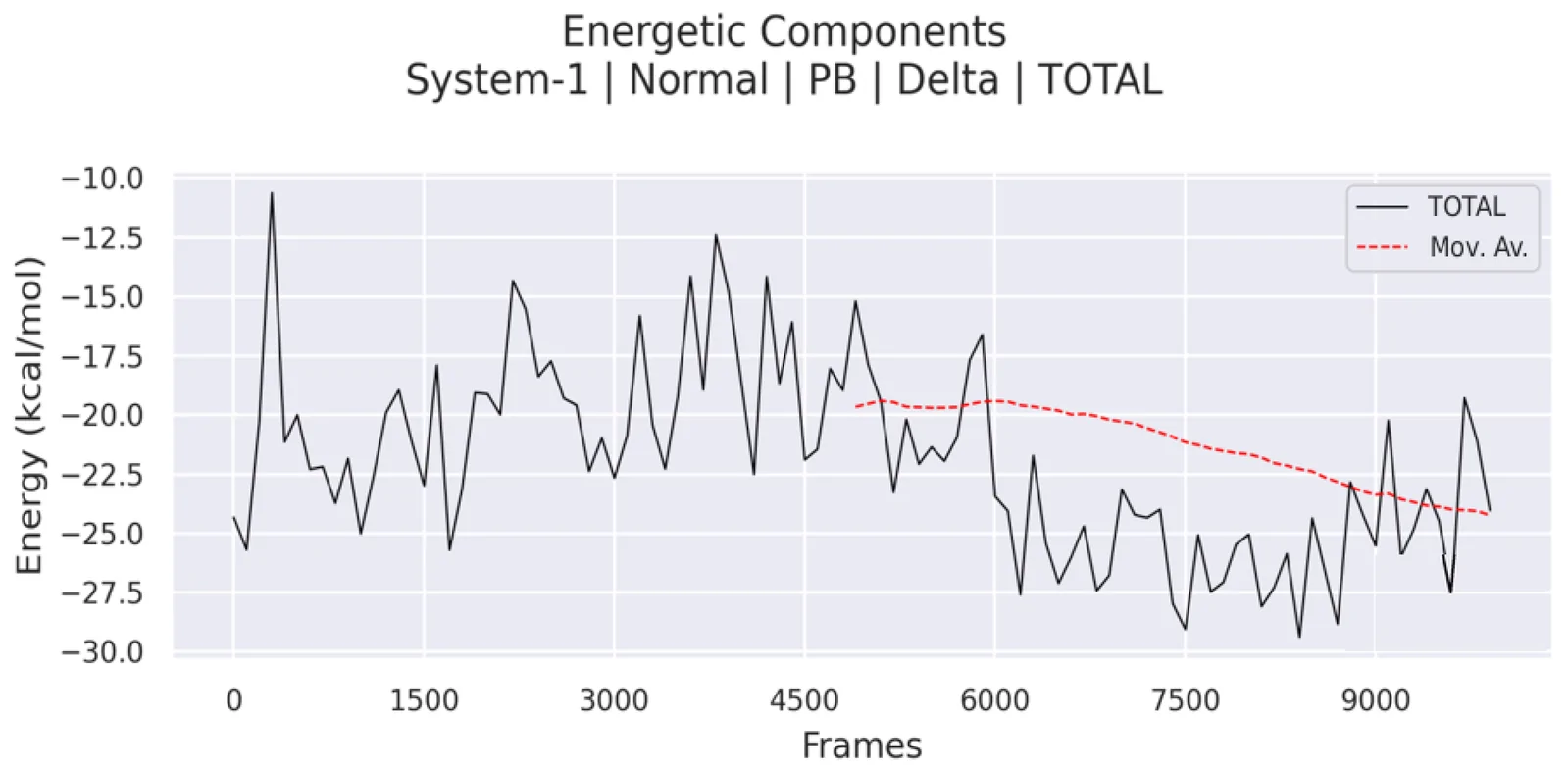
Frame-resolved MM/PBSA binding free energy along the production trajectory, illustrating the temporal stability of ligand binding.

Binding free energies extracted by the MM/GBSA and MM/PBSA formalisms converged on a strongly negative, and therefore thermodynamically favorable, interaction. The MM/GBSA estimate yielded ΔG_bind = −23.98 kcal·mol^−1^ (Figure 16), with the frame-resolved profile (Figure 17) demonstrating a stable energy plateau across the equilibrated portion of the trajectory. The independent MM/PBSA calculation produced an essentially concordant ΔG_bind of −21.95 kcal·mol^−1^ (Figure 18), with a similarly stable frame-by-frame trace (Figure 19). The 2 kcal·mol^−1^ discrepancy between the two implicit-solvent models is well within the expected range for end-point methods and reinforces, rather than questions, the spontaneous nature of complex formation.

**Figure 20:**
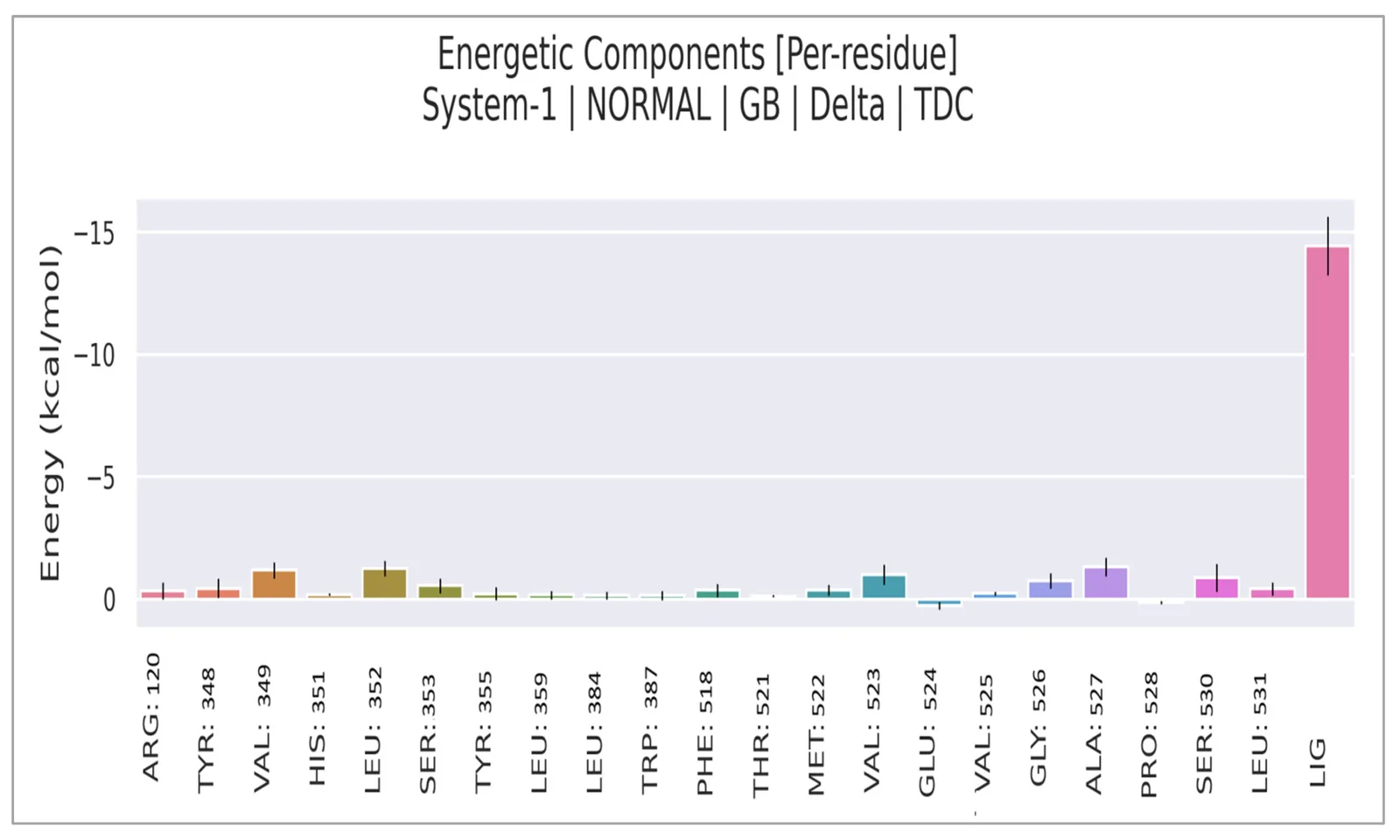
Per-residue MM/GBSA energy decomposition. Val349, Leu352, Ser353, Val523, Gly526, Ala527, and Ser530 are identified as the principal energetic contributors to ligand binding.

**Figure 21:**
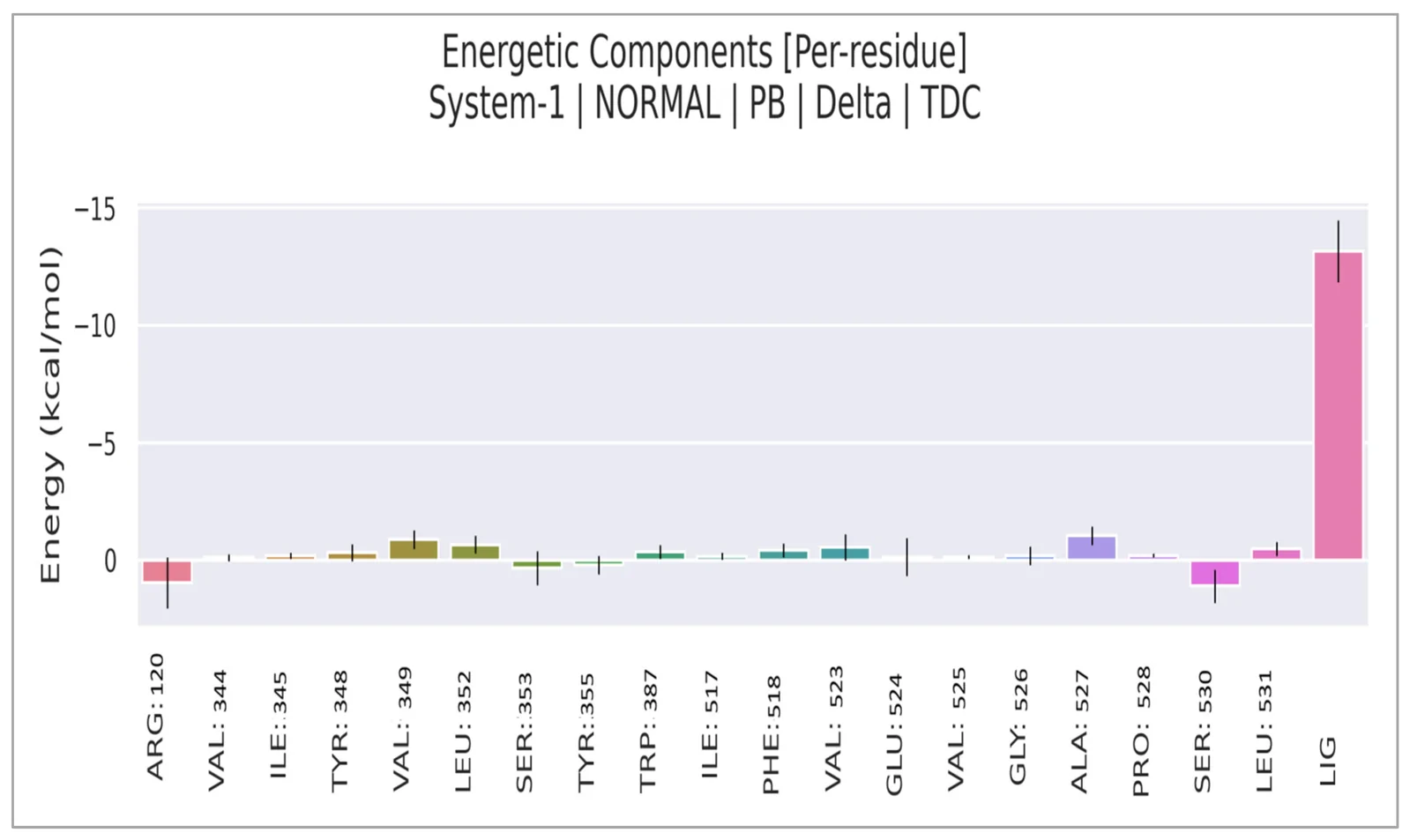
Per-residue MM/PBSA energy decomposition. Val349, Leu352, Val523, Ala527, and Leu531 emerge as the dominant stabilizing residues, in close agreement with the MM/GBSA result.

Per-residue energy decomposition unambiguously identified the principal energetic contributors. Within the MM/GBSA framework, Val349, Leu352, Ser353, Val523, Gly526, Ala527, and Ser530 emerged as dominant stabilizing residues (Figure 20), whereas the MM/PBSA decomposition emphasized Val349, Leu352, Val523, Ala527, and Leu531 (Figure 21). The remarkable convergence of the two implicit-solvent schemes around an essentially identical residue set and the perfect overlap of this set with the docking-derived contact map (Section 2.3) provides strong, internally consistent evidence that the predicted binding pocket is preserved throughout the simulation and that recognition is driven by hydrophobic packing within the COX-2 selectivity sub-pocket. A summary of the energetic decomposition is provided in Table 8.

### 2.7. In Silico ADMET Profiling of the Candidate Scaffolds

Affinity for the biological target is a necessary but insufficient criterion for advancing a candidate molecule; its pharmacokinetic behavior and safety margins weigh just as heavily on developability. Accordingly, the absorption, distribution, metabolism, excretion, and toxicity (ADMET) descriptors of the lead sesquiterpene (Z,Z,Z)-1,5,9,9-tetramethyl-1,4,7-cycloundecatriene were estimated and benchmarked against four structurally related molecules (cardenolide, germacrene D, 2-decyldecahydronaphthalene, and citronellyl isovalerate) using the pkCSM prediction server. The complete descriptor set is compiled in Table 9.

#### 2.7.1. Absorption

Predicted human intestinal absorption was uniformly high across the panel, extending from 92.40% for germacrene D to 100.00% for cardenolide, with the lead sesquiterpene absorbed at 96.61%. Caco-2 monolayer permeability stayed within a favorable range for every member of the series (log Papp of 1.203 to 1.541, expressed in units of 10^−6^ cm·s^−1^), (Z,Z,Z)-1,5,9,9-tetramethyl-1,4,7-cycloundecatriene returning 1.266. Predicted aqueous solubility tracked the hydrophobic hydrocarbon frameworks, declining from the moderately soluble citronellyl isovalerate (log S = −4.839 log mol·L^−1^) to the sparingly soluble germacrene D (log S = −7.780 log mol·L^−1^); the lead compound assumed an intermediate value (log S = −5.373 log mol·L^−1^).

For dermal permeability, (Z,Z,Z)-1,5,9,9-tetramethyl-1,4,7-cycloundecatriene (log Kp = −1.555 cm·h^−1^), 2-decyldecahydronaphthalene (log Kp = −1.429 cm·h^−1^), and citronellyl isovalerate (log Kp = −1.784 cm·h^−1^) all lay above the −2.5 cm·h^−1^ boundary that customarily marks poor skin transit, which supports their suitability for topical or transdermal routes. Cardenolide (log Kp = −2.702 cm·h^−1^) and germacrene D (log Kp = −2.668 cm·h^−1^) were instead predicted to cross skin poorly. Efflux-transporter behavior was heterogeneous: the lead sesquiterpene was the only P-glycoprotein (P-gp) substrate in the set, yet it inhibited neither P-gp I nor P-gp II. Inhibition was instead compound-specific, with cardenolide inhibiting P-gp I and germacrene D inhibiting P-gp II, which flags a risk of transporter-mediated drug–drug interactions restricted to these two molecules.

#### 2.7.2. Distribution

The steady-state volume of distribution (VDss) differed markedly across the series and pointed to divergent tissue partitioning. (Z,Z,Z)-1,5,9,9-tetramethyl-1,4,7-cycloundecatriene attained the highest value (log VDss = 0.629 log L·kg^−1^), followed by 2-decyldecahydronaphthalene (0.544 log L·kg^−1^) and germacrene D (0.427 log L·kg^−1^), all indicative of substantial extravascular partitioning, whereas cardenolide showed comparatively limited distribution (log VDss = −0.184 log L·kg^−1^). Plasma protein binding estimates placed cardenolide (Fu = 0.007) and germacrene D (Fu = 0.000) in the almost fully bound category, while the lead sesquiterpene preserved a much larger free fraction (Fu = 0.306), a property that helps sustain the pool of unbound, active drug.

All five molecules were predicted to penetrate the blood–brain barrier (log BB = 0.492 to 0.966). Their central nervous system (CNS) penetration index (log PS) ranged from −2.181 for the lead sesquiterpene and −2.138 for 2-decyldecahydronaphthalene at the low end to −0.884 for germacrene D at the high end, so central exposure is predicted to vary appreciably among the scaffolds.

#### 2.7.3. Metabolism and Excretion

Metabolic predictions indicated that (Z,Z,Z)-1,5,9,9-tetramethyl-1,4,7-cycloundecatriene, 2-decyldecahydronaphthalene, and citronellyl isovalerate are not handled by the major cytochrome P450 isoforms CYP2D6 or CYP3A4, whereas cardenolide and germacrene D were each predicted as CYP3A4 substrates. Inhibition of CYP2C19, CYP2C9, CYP2D6, and CYP3A4 was absent throughout the panel; the single inhibitory signal concerned CYP1A2, predicted for both (Z,Z,Z)-1,5,9,9-tetramethyl-1,4,7-cycloundecatriene and germacrene D. That liability warrants caution when either compound is combined with CYP1A2 substrates of narrow therapeutic index.

Predicted total clearance was greatest for citronellyl isovalerate (log CL = 1.491 log mL·min^−1^·kg^−1^), then 2-decyldecahydronaphthalene (1.420 log mL·min^−1^·kg^−1^), germacrene D (1.395 log mL·min^−1^·kg^−1^), and the lead sesquiterpene (1.088 log mL·min^−1^·kg^−1^), while cardenolide was eliminated most slowly (0.519 log mL·min^−1^·kg^−1^). No compound was predicted as a substrate of the renal organic cation transporter 2 (OCT2), which rules out OCT2-dependent renal interactions.

#### 2.7.4. Toxicity Profile

The toxicity screen was broadly favorable for the entire panel. Every compound was predicted to be non-mutagenic (negative Ames test), non-hepatotoxic, and inactive at the hERG I channel. Germacrene D was the only scaffold predicted to block the hERG II channel, confining a possible cardiotoxic concern to that molecule.

The predicted human maximum tolerated dose was highest for (Z,Z,Z)-1,5,9,9-tetramethyl-1,4,7-cycloundecatriene (0.987 log mg·kg^−1^·day^−1^), ahead of citronellyl isovalerate (0.625), 2-decyldecahydronaphthalene (0.497), cardenolide (−0.376), and germacrene D (−0.528). Predicted acute oral toxicity in rats (LD50) remained within an acceptable interval for the whole series (1.634 to 2.489 mol·kg^−1^), the lead sesquiterpene registering 2.316 mol·kg^−1^. Predicted chronic oral toxicity (LOAEL) spanned 1.258 log mg·kg^−1^·day^−1^ for the lead compound to 2.471 log mg·kg^−1^·day^−1^ for citronellyl isovalerate.

Skin sensitization was predicted for four of the five lipophilic hydrocarbons, including the lead sesquiterpene, a characteristic often seen with volatile sesquiterpenoids that can be addressed through appropriate galenic formulation. Environmental descriptors (*Tetrahymena pyriformis* growth inhibition and minnow toxicity) pointed to a low-to-moderate ecological footprint; notably, (Z,Z,Z)-1,5,9,9-tetramethyl-1,4,7-cycloundecatriene displayed the lowest aquatic toxicity toward minnow (0.674 log mM), contrasting with the marked aquatic toxicity predicted for cardenolide (−2.459 log mM) and germacrene D (−1.691 log mM).

## 3. Integrative Discussion

The present multiscale computational and physicochemical investigation provides a rigorous atomistic rationale for the ethnomedical utilization of *Corymbia citriodora* essential oil in the management of inflammatory pathologies [22,23,24]. By deploying a sequential funneling strategy from macroscopic phytochemical profiling to quantum electronic characterization, and finally to dynamic thermodynamic assessment this study systematically dissects the molecular determinants governing the targeted inhibition of cyclooxygenase-2 (COX-2) by plant-derived volatile scaffolds [25,26].

The chromatographic analysis juxtaposing conventional steam distillation (SD) and microwave-assisted steam distillation (MSD) underscores a crucial fractionation dichotomy, consistent with prior reports that C. citriodora oils are dominated by citronellal and related monoterpenoids while also containing minor sesquiterpenes [24,27]. While the dielectric heating mechanism intrinsic to MSD preferentially enriched polar oxygenated monoterpenes, most notably citronellal (48.41%), our high-throughput virtual screening campaign revealed an inverse correlation between relative abundance and target affinity, in line with virtual-screening work where low-abundance terpenoids emerge as preferred COX-2 binders [26,28]. It was the quantitatively minor hydrocarbon sesquiterpene fraction that dominated the top-scoring docked poses, as also observed for sesquiterpenoids from *Pogostemon herba* and *Zingiber* species [28,29]. The lead compound, (Z,Z,Z)-1,5,9,9-tetramethyl-1,4,7-cycloundecatriene, alongside structurally analogous C_15_ macrocycles, exhibited superior geometric complementarity to the deep, predominantly hydrophobic cyclooxygenase substrate channel, consistent with other hydrophobic COX-2 inhibitors [30]. This topological congruence highlights a classic paradigm in phytopharmacology wherein macroscopic bioactivity may be driven by highly potent, low-abundance constituents acting either independently or via synergistic “entourage” mechanisms with the solvent-like bulk monoterpenes [22,26].

At the level of receptor–ligand recognition, the binding architecture of the lead sesquiterpene elegantly exploits the distinct topological features of the COX-2 active site, including the hydrophobic side pocket that underlies COX-2 selectivity [30,31,32]. The two-dimensional interaction map and subsequent trajectory-averaged per-residue energy decompositions robustly identified Val523 as a critical anchoring point, in agreement with mutagenesis and free-energy studies that pinpoint Val523 (Ile523 in COX-1) as a key selectivity hotspot [30,31,32]. The substitution of isoleucine (found in COX-1) with the less sterically demanding valine at position 523 is the primary structural determinant of COX-2 selectivity, as it effectively opens a lateral hydrophobic pocket [30,31,32]. The rigid, eleven-membered saddle-like conformation of (Z,Z,Z)-1,5,9,9-tetramethyl-1,4,7-cycloundecatriene facilitates deep insertion into this lateral sub-pocket, engaging in pervasive π-alkyl and van der Waals contacts with Val349, Leu352, and Ala527 residues repeatedly implicated in hydrophobic recognition of selective COX-2 inhibitors [32,33]. Consequently, the ligand acts as a highly selective steric wedge that occludes the upstream migration of arachidonic acid toward the peroxidase catalytic core, a mechanism consistent with structural and kinetic analyses of time-dependent COX-2 inhibition [30,31,32].

Quantum chemical characterization at the B3LYP/3-21G level within a CPCM solvation framework further substantiated the pharmacological viability of this scaffold, mirroring DFT-based drug-design work where large HOMO–LUMO gaps and high hardness correlate with thermodynamic stability and reduced nonspecific reactivity [34,35,36]. The substantial HOMO–LUMO gap (6.12 eV) and high chemical hardness (η = 3.06 eV) portray a kinetically stable, unreactive macrocycle, in line with other B3LYP-optimized bioactive molecules [34,36]. The absence of localized electrophilic or nucleophilic warheads, as corroborated by the smoothly distributed molecular electrostatic potential (ESP) surface, indicates that the ligand functions exclusively as a non-covalent, reversible inhibitor, paralleling noncovalent COX-2 scaffolds identified in pharmacophore, QSAR, and docking pipelines [25,32]. This is a highly desirable trait, as it bypasses the idiosyncratic hepatotoxicities and immunogenic responses frequently associated with xenobiotics that covalently adduct to off-target hepatic or plasma proteins via Michael addition or reactive oxidative intermediate formation [37].

The temporal and thermodynamic stability of this non-covalent assembly was unambiguously validated by the 100 ns molecular dynamics simulation, consistent with MD-based assessments of COX-2 inhibitor stability [25,26,28]. The exceptionally low backbone RMSD (0.17 ± 0.02 nm) and stable radius of gyration strongly suggest that the ligand does not induce allosteric frustration or global denaturation, echoing previous MD studies where stable COX–ligand complexes preserve overall fold [25,28,32]. Instead, principal component analysis (PCA) and the computed Gibbs free energy landscape (FEL) mapped a highly restricted conformational space, indicating that the complex is rapidly funneled into a deep thermodynamic basin behavior analogous to other high-affinity COX-2 complexes characterized by narrow FEL basins [25,26,38]. The corroborating end-point MM/GBSA (-23.98 kcal·mol^−1^) and MM/PBSA (-21.95 kcal·mol^−1^) calculations confirm that the binding event is overwhelmingly driven by enthalpic desolvation and favorable van der Waals packing, characteristic of classical hydrophobic effect–driven ligand entrapment within the COX-2 core [25,26,28].

Finally, the in silico ADMET profiling underscores the translational tractability of the sesquiterpene candidate, paralleling multi-parameter evaluations used for other COX-2–directed scaffolds [25,26,33]. The high computed intestinal absorption (96.61%) and favorable Caco-2 permeability metrics align with the scaffold’s lipophilicity (logP ~ 5.04), comparable to other lipophilic COX-2 inhibitors with good oral bioavailability [25,30,39]. While the molecule possesses optimal topological dimensions for membrane traversal, including potential blood–brain barrier penetration (log BB = 0.492) for neuroinflammatory applications [40], the specific flag for CYP1A2 inhibition necessitates cautious clinical consideration, as in other candidates where cytochrome P450 liabilities threaten drug–drug interactions [41]. CYP1A2 mediates the biotransformation of several narrow-therapeutic-index therapeutics, implying a risk of pharmacokinetic drug–drug interactions. Furthermore, the predicted skin sensitization liability is structurally concordant with the lipophilic nature of unsaturated terpenoids and parallels sensitization alerts seen for other terpenoid COX-2 ligands [42]. Nonetheless, the overall toxicological window marked by the absence of hERG-mediated cardiotoxicity, AMES mutagenicity, and hepatotoxicity presents a fundamentally safer profile than legacy synthetic coxibs, in line with recent virtual-screening and pharmacokinetic studies that identify natural-product COX-2 inhibitors with reduced cardiovascular and hepatic risk [25,32,33], thereby validating the repurposing of C. citriodora sesquiterpenes as next-generation anti-inflammatory pharmacophores [22,23,24].

Crucially, expanding our virtual screening campaign across a multi-target panel encompassing human COX-2 (5KIR/3LN1), COX-1 (4COX), and iNOS (3E7G) demonstrates a poly-pharmacological mechanism for C. citriodora constituents. The lead sesquiterpene (Z,Z,Z)-1,5,9,9-tetramethyl-1,4,7-cycloundecatriene demonstrated consistently high affinity across COX-2 (−8.0 to −8.2 kcal/mol) and iNOS (−6.8 kcal/mol). Dual inhibition of COX-2 and iNOS is a well-established therapeutic target in acute and chronic inflammation, as both enzymes are co-induced by pro-inflammatory cytokines and act in synergy to promote hyperalgesia and tissue injury through PGE_2_ and nitric oxide (NO) generation. Simultaneous attenuation of COX-2 and iNOS pathways offers superior anti-inflammatory efficacy while minimizing adverse effects.

## 4. Materials and Methods

### 4.1. Plant Material and Essential Oil Extraction

Healthy mature *Corymbia citriodora* trees growing on the grounds of the National Higher School of Agronomy (ENSA), Algeria (36.7222384° N, 3.1497033° E), provided the fresh foliage used in this work, which was harvested during the period of active vegetative growth. The harvested material was allowed to dry in the shade at ambient laboratory temperature, and the resulting dry leaves were then ground. Two parallel extraction approaches were applied to the powdered leaf biomass: classical steam distillation (SD) and steam distillation enhanced by microwave irradiation (MSD). In the SD procedure, a 200 g portion of plant matter was distilled for 120 min in a Clevenger-type apparatus (Schott Duran, Schott AG, Mainz, Germany) at a steam flow of 10 mL·min^−1^ (power consumption: 1000 W mantle, ~2.0 kWh total energy). The MSD protocol employed an identical mass of plant material (200 g) irradiated at 500 W for 20 min under an identical steam flow rate (~0.167 kWh total energy). The 20 min duration for MSD was established based on kinetic optimization showing complete extraction yield due to rapid microwave-induced glandular rupture. The recovered hydrodistillates were subsequently dehydrated over anhydrous sodium sulfate (Sigma-Aldrich, St. Louis, MO, USA), transferred into amber glass vials, and kept at 4 °C pending analysis.

### 4.2. Gas Chromatography–Mass Spectrometry–Mass Spectrometry Analysis

Compositional characterization of the essential oil relied on a Shimadzu GCMS-TQ8040 NX system coupling gas chromatography to tandem mass spectrometry (GC-MS/MS) by means of a triple quadrupole mass analyzer, the unit being equipped with an AOC-30/20i autosampler (Shimadzu Corporation, Kyoto, Japan). Sample preparation prior to injection consisted of preparing a 10% (*v*/*v*) solution by combining 100 µL of essential oil with 900 µL of chromatography-grade hexane (Sigma-Aldrich, St. Louis, MO, USA). A 1 µL portion of this solution was then introduced into the injector (held at 250 °C) under split mode operating at a 1:100 ratio.

The chromatographic resolution of components was performed on an RTX-5SILMS fused-silica capillary column (length: 30 m; internal diameter: 0.25 mm; film thickness of stationary phase: 0.25 µm). Helium served as the carrier gas, supplied at a constant rate of 1.20 mL/min, equivalent to a linear velocity of 39.4 cm/s. The oven temperature program initiated with a 4 min hold at 35 °C, after which the temperature was ramped at 10 °C/min up to 240 °C; a second ramp of 8 °C/min then drove the column to its final temperature of 300 °C, which was sustained for 8 min.

Mass spectrometric detection was carried out under electron ionization (EI) at 70 eV, with mass acquisition spanning 30–500 m/z. Temperatures of 200 °C and 250 °C were maintained for the ion source and the transfer line, respectively, while a solvent delay of 3.00 min was applied.

Compound identification rested upon matching electron ionization mass spectra against NIST 20 (National Institute of Standards and Technology, Gaithersburg, MD, USA), Wiley 12 (John Wiley & Sons, Hoboken, NJ, USA), and FFNSC 4 spectral databases (version 4.0, Shimadzu Corporation, Kyoto, Japan) alongside comparing calculated Kovats retention indices (RI, derived relative to C_7_–C_40_ n-alkanes) with literature values (ΔRI<10). Principal constituents (citronellal, citronellol, isopulegol, β-caryophyllene) were further confirmed using commercial reference standards (Sigma-Aldrich, St. Louis, MO, USA). Quantitative compositional variations between SD and MSD extractions were evaluated descriptively through relative GC peak area normalization.

### 4.3. Target Protein Preparation

**Figure 22:**
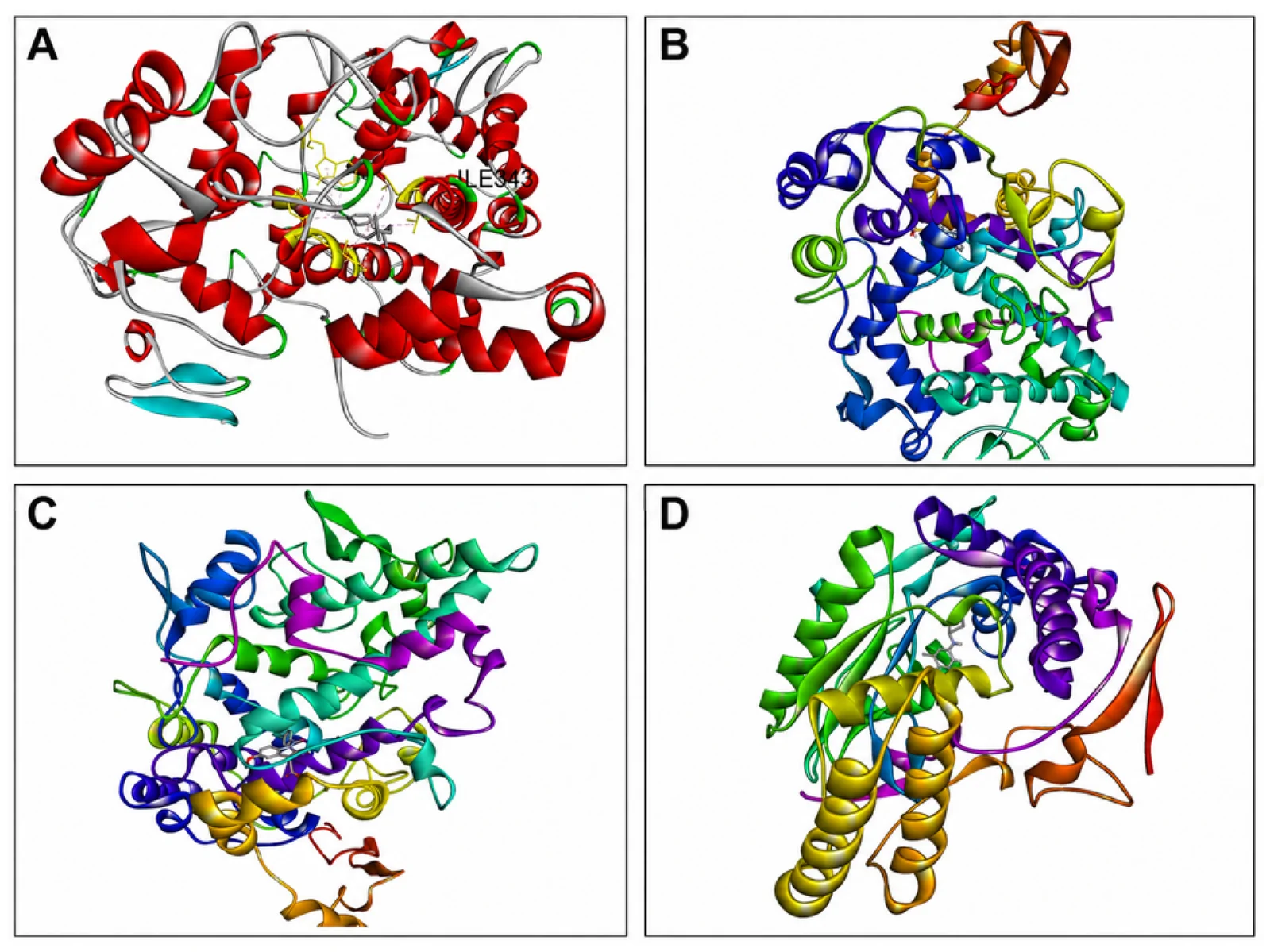
Three-dimensional ribbon representations of the target protein structures: (**A**) Human cyclooxygenase-2 bound to rofecoxib (PDB ID: 5KIR), colored by secondary structure elements (alpha-helices in red, beta-sheets in cyan, and loops in grey/green), showing the membrane-binding domain (MBD), catalytic core, substrate channel, and heme cofactor; (**B**) Human cyclooxygenase-2 bound to celecoxib (PDB ID: 3LN1); (**C**) Ovine cyclooxygenase-1 bound to indomethacin (PDB ID: 4COX); and (**D**) Human inducible nitric oxide synthase (iNOS) bound to AR-C95791 (PDB ID: 3E7G), with structures (**B**–**D**) rendered with a rainbow color gradient along the polypeptide chains from the N-terminus (blue) to the C-terminus (red). Bound ligands and cofactors in the active sites are highlighted within each respective catalytic channel.

Atomic coordinates of four key inflammatory targets were retrieved from the RCSB Protein Data Bank: human COX-2 bound to rofecoxib (PDB: 5KIR; 2.70 Å resolution), COX-2 bound to celecoxib (PDB: 3LN1; 2.40 Å), ovine COX-1 bound to indomethacin (PDB: 4COX; 3.00 Å), and human inducible nitric oxide synthase (iNOS) bound to AR-C95791 (PDB: 3E7G; 2.20 Å). All structures were processed by removing water molecules, co-crystallized heteroatoms, and co-factors prior to grid generation (Figure 22) [43,44]. Preprocessing of the structure was carried out within BIOVIA Discovery Studio together with AutoDockTools (ADT v1.5.7) and involved deletion of crystallographic waters, the bound ligand, and extraneous heteroatoms, in keeping with conventional docking preparation pipelines. Hydrogens of polar character were appended, Gasteiger-type partial charges were attributed, and apolar hydrogens were merged into their parent heavy atoms. The receptor thus prepared was saved in PDBQT format [45,46,47]. Delineation of the binding cavity proceeded from two complementary perspectives: first, by direct visual analysis of the contact pattern between rofecoxib and the enzyme, and second, by automated cavity prediction performed through the PrankWeb server (https://prankweb.cz/, accessed in 23 April 2026), which uses a machine-learning algorithm [48,49,50]. The agreement of these two independent strategies allowed a reliable demarcation of the catalytic site for the docking experiments to follow, in accordance with current recommendations advocating the joint use of experimentally observed inhibitor binding modes and contemporary cavity-prediction tools for defining the COX-2 active region [43,44,49].

### 4.4. Ligand Library and Preparation

For all sixty-three compounds detected in the essential oil profile, three-dimensional structural files were retrieved as SDF entries from the PubChem repository, with each retrieval guided by the corresponding CID accession number [51,52]. Once collected, every molecular structure went through an energy-minimization step relying on the MMFF94 force field [53] within the Open Babel software (v3.1.1); the rotational degrees of freedom were thereafter defined for each molecule, and the energy-minimized geometries were finally exported under the PDBQT file extension [54,55,56].

### 4.5. Molecular Docking Protocol

Molecular docking computations were performed with the aid of AutoDock Vina (v1.2.0), launched from within the PyRx graphical environment (v0.8). A search box measuring 25.90 Å × 26.64 Å × 29.96 Å was set up so as to encompass the rofecoxib-occupied pocket, with its geometric center placed at the Cartesian coordinates (x = 24.75, y = 40.43, z = 6.33). The exhaustiveness parameter was held at a value of 8, while nine alternative binding orientations were generated per ligand. Among these, the orientation chosen for downstream analysis was the one that simultaneously displayed the lowest Vina-derived binding affinity score and a satisfactory geometric placement within the catalytic cleft. For protocol validation, the rofecoxib ligand that had been co-crystallized with the protein in its original structure was redocked into its native pocket, and the predicted orientation was superposed on the experimentally resolved coordinates. Subsequently, both planar and three-dimensional representations of the protein–ligand interactions were drawn in BIOVIA Discovery Studio Visualizer (version 21.1.0), allowing visualization of hydrogen bonds, π-alkyl contacts, π-sigma interactions, alkyl interactions, as well as van der Waals contacts [18,54,55,57,58,59].

Docking protocols were rigorously validated by extracting each native co-crystallized ligand (rofecoxib for 5KIR, celecoxib for 3LN1, indomethacin for 4COX, and AR-C95791 for 3E7G) and re-docking it into its respective binding cavity. Alignment between predicted and experimental poses was quantified via Root Mean Square Deviation (RMSD), with values <2.0 Å signifying valid protocols.

### 4.6. Density Functional Theory Calculations

The lead sesquiterpene was subjected to quantum mechanical analysis based on density functional theory (DFT), with all calculations executed in the Gaussian 09 software package (Revision A.02) [60,61,62]. An unconstrained optimization of the molecular geometry, with no symmetry conditions imposed, was performed at the B3LYP functional level in combination with the 3-21G basis set [63,64]. The influence of the surrounding medium was modeled implicitly using the conductor-like polarizable continuum model (CPCM), embedded within the self-consistent reaction field (SCRF) approach [60,65,66,67,68]. Confirmation that the optimized structure represented a genuine minimum on the potential energy surface was provided by the absence of any negative vibrational frequency. From the resulting wavefunction, several quantum descriptors were extracted, namely the energies of the highest occupied (HOMO) and lowest unoccupied (LUMO) molecular orbitals, the energy difference between them, the dipole vector, and the molecular electrostatic potential (ESP) surface. A series of global reactivity parameters was then calculated through Koopmans’ theorem including the ionization potential (I), the electron affinity (A), the electronegativity (χ), the chemical potential (μ), the chemical hardness (η), the softness (S), and the electrophilicity index (ω) following the customary mathematical relations χ = (I + A)/2, μ = −χ, η = (I − A)/2, S = 1/η, and ω = μ^2^/(2η) [69].

### 4.7. Molecular Dynamics Simulation

Molecular dynamics (MD) simulations carried out at the atomistic level for COX-2 receptor bound to its ligand were carried out within GROMACS (v2023.2), employing the CHARMM27 force field for parameterization [70,71,72]. The topology of the sesquiterpene molecule was specifically built using the SwissParam online tool (v2011), which guarantees its consistency with the CHARMM force-field family [73,74,75]. Following parameterization, the protein–ligand complex was placed at the center of a cubic periodic box and surrounded by explicit water molecules described by the TIP3P model, with the box edge separated from any atom of the solute by no less than 1.0 nm [70,76]. Net charge neutrality of the simulation system was achieved by introducing the appropriate number of counter-ions [77].

The system was first relaxed energetically by means of the steepest-descent minimization protocol, allowed to run for up to 50,000 iterations, and terminated as soon as the largest atomic force fell under the threshold of 100 kJ·mol^−1^·nm^−1^. Two sequential equilibration phases followed the minimization step: an initial 500 ps trajectory under the NVT ensemble at 300 K, where the temperature was maintained by the V-rescale thermostat, and a subsequent 2 ns NPT trajectory at 300 K and 1 bar in which pressure regulation was achieved through the Parrinello–Rahman barostat operated under isotropic coupling (with a coupling time constant τ_*p* = 2 ps) [72,73]. Once equilibration was completed, a 100 ns production trajectory was generated applying periodic boundary conditions together with a time step of 2 fs. Covalent bonds involving hydrogen atoms were held rigid through the LINCS constraint scheme, while long-range electrostatic interactions extending beyond the cutoff range were handled by means of the smooth particle mesh Ewald (PME) method [70,78]. Standard structural and dynamic descriptors derived from the simulation trajectory including root-mean-square deviation (RMSD), root-mean-square fluctuation (RMSF), radius of gyration (Rg), and solvent-accessible surface area (SASA), and the temporal evolution of hydrogen bonds were calculated using the built-in analysis programs of GROMACS [73,77,79,80].

### 4.8. Principal Component Analysis and Free Energy Landscape

Mapping of the dominant collective motions of the protein was performed by computing and diagonalizing the mass-weighted covariance matrix built from the trajectory of Cα atomic positions, a step achieved through the gmx covar and gmx anaeig commands. The eigenvectors and corresponding eigenvalues issuing from this decomposition were subsequently imported into the Bio3D package executed in the R programming environment, and the largest-amplitude conformational deviations along the principal modes were rendered as porcupine plots within PyMOL (v2.5). To construct the Gibbs free energy landscape (FEL), the joint probability distribution generated by the first and second principal components was processed through the gmx sham routine, and the resulting energy surface was plotted in Python (v3.10) with the Matplotlib library (v3.7). The pattern of correlated and anti-correlated motions among residue pairs was further explored through the dynamic cross-correlation matrix (DCCM), which was computed in Bio3D after the GROMACS trajectory file had been converted into the .dcd format with the help of VMD [38,81,82,83].

### 4.9. Binding Free Energy and Per-Residue Decomposition

Approximations of the binding free energy were derived through end-point methods, namely the Molecular Mechanics combined with Generalized Born and Surface Area continuum solvation (MM/GBSA) procedure, as well as its Poisson–Boltzmann counterpart (MM/PBSA), both supported by the g_mmpbsa software (v1.6) [84]. The energetic evaluations were carried out on a representative ensemble of frames extracted from the converged portion of the production trajectory, applying the classical thermodynamic relationship ΔG_bind = G_complex − (G_protein + G_ligand) [85]. Within the same computational pipeline, the contribution of each individual residue to the overall binding affinity was isolated through per-residue energy decomposition, allowing the major amino-acid contributors to be highlighted; this analysis offered a direct mechanistic bridge between the contacts detected at the docking stage and the time-averaged energetic landscape obtained from the simulation [75,85,86,87,88].

### 4.10. In Silico ADMET Profiling

The pharmacokinetic and toxicological profiles of the top five virtual screening candidate compounds (Z,Z,Z)-1,5,9,9-tetramethyl-1,4,7-cycloundecatriene, cardenolide, germacrene D, 2-decyldecahydronaphthalene, and citronellyl isovalerate were evaluated using a fully computational approach via the pkCSM web server (https://biosig.lab.uq.edu.au/pkcsm/) (accessed on 10 July 2026), a predictive platform that utilizes graph-based molecular signatures to establish correspondences between chemical architecture and ADMET-relevant properties [89]. Each candidate compound was submitted in its canonical SMILES format to generate predicted outputs spanning the four classical pharmacokinetic compartments absorption, distribution, metabolism, and excretion alongside a comprehensive panel of toxicological endpoints.

Within the absorption compartment, the evaluated descriptors included water solubility (logS), permeability across Caco-2 cell monolayers ($\log P_{\mathrm{app}}$), the percentage of compound absorbed in the human gut, transcutaneous permeability ($\log K_{p}$), and interactions with P-glycoprotein, assessed both as substrate recognition and as inhibitory potency toward the P-gp I and P-gp II transporters [90,91,92,93]. The distribution-related outputs gathered the volume of distribution at steady state (${\mathrm{VD}}_{\mathrm{ss}}$), the unbound plasma fraction ($F_{u}$), blood–brain barrier permeability (logBB), and central nervous system penetration index (logPS). Predictions associated with metabolism focused on whether the molecules behaved as substrates for the cytochrome P450 isoforms CYP2D6 and CYP3A4, as well as their capacity to inhibit five major isoenzymes of the same superfamily: CYP1A2, CYP2C19, CYP2C9, CYP2D6, and CYP3A4 [91,92]. Excretion was characterized by total body clearance (logCL) and recognition as a substrate for the renal organic cation transporter 2 (OCT2).

The toxicological panel included a comprehensive series of endpoints: Ames mutagenicity testing, predicted maximum tolerated dose in humans, blockade of cardiac hERG I and II potassium channels, median lethal dose by oral route in rats (${\mathrm{LD}}_{50}$), chronic toxic dose threshold expressed as LOAEL, hepatic toxicity potential, skin sensitization tendency, toxicity toward the protozoan *Tetrahymena pyriformis*, and acute toxicity in minnows. In parallel with these biological predictions, fundamental physicochemical properties including molecular weight, octanol-to-water partition coefficient (logP), number of freely rotatable bonds, counts of hydrogen-bond donors and acceptors, and topological polar surface area (TPSA) were estimated to verify compliance with Lipinski’s rule of five and general criteria for drug-likeness [94].

### 4.11. Quantitative Compositional Assessment

The quantitative compositional comparison between Steam Distillation (SD) and Microwave-Assisted Steam Distillation (MSD) was evaluated descriptively. Relative percentage concentrations of individual constituents were calculated via GC peak area normalization, assuming equal mass response factors across all volatile components.

## 5. Conclusions

This study presents an integrated phytochemical and multi-target computational assessment of *Corymbia citriodora* essential oil as a source of anti-inflammatory leads. GC–MS/MS profiling established citronellal as the dominant volatile constituent. Structure-based virtual screening across COX-2, COX-1, and iNOS targets validated by native ligand re-docking identified a hydrocarbon sesquiterpene cluster, with (Z,Z,Z)-1,5,9,9-tetramethyl-1,4,7-cycloundecatriene acting as a potent multi-target lead. Comparative DFT calculations confirmed that this sesquiterpene possesses high kinetic stability (ΔE=6.12 eV; η=3.06 eV), operating via non-covalent hydrophobic shape complementarity. All-atom MD simulations and MM/GBSA/PBSA binding free energies (−23.98 and −21.95 kcal/mol) confirmed the structural and thermodynamic stability of the COX-2 complex, anchored at key residues including Val523. Combined with favorable predictive ADMET properties, these findings provide a residue-resolved blueprint for developing *C. citriodora*-derived sesquiterpenes as novel multi-target anti-inflammatory therapeutics.

While this study provides a multiscale computational framework for identifying *C. citriodora* constituents as COX-2 inhibitors, several methodological limitations must be acknowledged. First, the study relies entirely on in silico predictions. Docking scores, MD trajectories, MM/PBSA/GBSA binding free energies, and pkCSM ADMET outputs are theoretical estimations that cannot replace empirical biological validation. Computational ADMET algorithms cannot fully simulate complex physiological factors such as intestinal microbiota metabolism, first-pass hepatic transformation, or tissue distribution.

Second, static docking relied on a single crystal structure (PDB: 5KIR). Future virtual screening workflows should incorporate ensemble docking across multiple COX-2 structures (e.g., 1CX2, 3NT1) to account for target backbone flexibility. For quantum calculations, the B3LYP/3-21G level provided a preliminary electronic overview; subsequent studies will employ larger basis sets containing polarization and diffuse functions (e.g., B3LYP/6-311++G(d,p)). Furthermore, execution of independent triplicate MD trajectories will further reinforce statistical reproducibility.

Future work must actively bridge these theoretical findings with experimental testing. The immediate next step involves in vitro screening of isolated or synthesized (Z,Z,Z)-1,5,9,9-tetramethyl-1,4,7-cycloundecatriene using colorimetric COX-1 and COX-2 inhibition assays to determine exact IC_50_ values and establish isoform selectivity ratios. Cell-based validation using LPS-stimulated RAW 264.7 macrophages will quantify downstream reductions in PGE_2_, iNOS, and pro-inflammatory cytokines. Given the complex chemistry of essential oils, multi-target CADD studies evaluating dual inhibition against 5-LOX, iNOS, and NF-κB pathways are also recommended.

Finally, addressing predicted pharmacokinetic liabilities specifically high lipophilicity (logP ~ 5.04) and potential skin sensitization will require galenic interventions. Nano-emulsions, solid lipid nanoparticles, or liposomal formulations could enhance aqueous solubility, control systemic release, and minimize localized skin irritation for topical applications.

## Figures and Tables

**Table 1:** Chemical composition of *Corymbia citriodora* essential oil obtained by steam distillation (SD) and microwave-assisted steam distillation (MSD).

No.	Compound	RI ^a^	RT (min)	Area (%) SD	Area (%) MSD
1	Isobutyric acid, isobutyl ester	894	5.812	0.05	0.08
2	α-Pinene	914	6.037	0.43	0.13
3	Thujene	948	6.395	0.12	0.09
4	*β*-Pinene	953	6.452	1.40	0.68
5	*β*-Myrcene	959	6.512	0.13	0.08
6	Isobutyl isovalerate	971	6.640	-	0.04
7	1-Methylbutyl butyrate	976	6.687	-	0.07
8	Propanoic acid, 2-methyl-, 2-methylbutyl ester	978	6.715	-	0.04
9	o-Cymene	990	6.844	0.06	-
10	1,8-Cineole	998	6.922	2.20	1.53
11	5-Heptenal, 2,6-dimethyl-	1011	7.051	1.45	0.68
12	*γ*-Terpinene	1019	7.128	0.21	0.17
13	trans-Isolimonene	1030	7.239	0.15	0.05
14	(+)-4-Carene	1044	7.376	0.08	0.19
15	1,5-Cyclooctadiene, 1,2-dimethyl-	1048	7.421	0.09	0.57
16	Linalool	1052	7.455	0.26	-
17	cis-Rose oxide	1062	7.559	0.51	0.14
18	Citronellol (isomer)	1077	7.700	0.95	0.32
19	Citronellal	1102	7.953	38.24	48.41
20	Isopulegol	1114	8.073	8.56	6.46
21	neo-Isopulegol	1123	8.162	2.02	1.38
22	Isopulegol (isomer)	1133	8.258	0.65	0.62
23	α-Terpineol	1143	8.365	0.41	0.32
24	(R)-2,6-Dimethylhept-5-enoic acid	1149	8.425	0.12	0.09
25	2-(1-Hydroxy-1-methylethyl) cyclohexanol	1154	8.468	0.07	-
26	Citronellol	1166	8.594	15.43	15.16
27	Geraniol	1188	8.815	0.22	0.04
28	Pinol	1192	8.853	0.18	0.15
29	p-Menth-8-en-3-ol, acetate	1210	9.041	0.30	0.09
30	2-Octen-4-ol, 2-methyl-	1217	9.118	0.55	0.29
31	Citronellic acid	1241	9.374	5.00	2.50
32	2-Methoxy-4-vinylphenol	1250	9.477	1.20	0.74
33	Cardenolide	1259	9.567	0.29	0.16
34	Citronellyl isovalerate	1278	9.775	7.04	9.19
35	Eugenol	1290	9.900	0.27	0.19
36	p-Menthane-3,8-diol, cis-1,3,trans-1,4-	1292	9.930	0.17	0.16
37	(1S,2R,5R)-2-(2-Hydroxypropan-2-yl)-5-methylcyclohexanol	1300	10.014	1.65	3.64
38	Geranyl acetate	1305	10.075	0.07	0.29
39	p-Menthane-3,8-diol (isomer a)	1310	10.126	0.07	-
40	2-Cyclopenten-1-one, 3-methyl-2-(2-pentenyl)-	1328	10.337	1.60	0.51
41	2-Cyclopenten-1-one, 3-methyl-2-(2-pentenyl)-	1328	10.338	-	0.71
42	p-Menthane-3,8-diol (isomer b)	1352	10.608	0.24	0.14
43	β-Caryophyllene	1363	10.734	2.14	1.44
44	1,4,7-Cycloundecatriene, 1,5,9,9-tetramethyl-	1396	11.117	0.11	0.12
45	1,1,4,7-Tetramethyldecahydro-1H-cyclopropa[e]azulene	1401	11.171	0.21	0.13
46	Germacrene D	1419	11.379	0.11	0.04
47	Germacrene D	1419	11.379	-	0.22
48	Isocaryophyllene	1427	11.481	0.03	0.05
49	Bicyclogermacrene	1432	11.539	0.27	0.47
50	1,5-Cyclodecadiene, 1,5-dimethyl-8-(1-methyl)	1493	12.252	-	0.03
51	(−)Spathulenol	1508	12.430	1.34	0.44
52	Isolongifolol	1515	12.512	1.56	0.44
53	Guaiol	1525	12.640	0.10	0.04
54	Farnesol (isomer)	1529	12.680	0.13	0.07
55	Ledol	1535	12.755	0.09	0.05
56	12-Oxabicyclo compound	1539	12.807	0.11	0.03
57	(−)Spathulenol (isomer)	1557	13.012	0.17	0.06
58	4,8-Dimethylenebicyclo[7.2.0]undecane deriv.	1563	13.082	0.09	-
59	1H-Cycloprop[e]azulen-7-ol, decahydro-	1577	13.257	0.11	-
60	7-Isopropyl-4,10-dimethylenecyclodecene	1590	13.408	0.13	-
61	Naphthalene, 2-decyldecahydro-	1635	13.968	0.10	0.10
62	4,8,13-Cyclotetradecatriene-1,3-diol	1876	16.635	0.50	0.14
63	(−)Isolongifolol	1891	16.805	0.11	-
Total identified				99.85	99.97
Chemical class summary					
Hydrocarbon monoterpenes				2.58	1.39
Oxygenated monoterpenes				88.27	93.23
Hydrocarbon sesquiterpenes				2.66	2.34
Oxygenated sesquiterpenes				3.72	1.13
Esters				0.05	0.23
Others				2.68	1.65

*^a^ Retention index calculated on an apolar column (DB-5ms or equivalent), relative to a homologous series of n-alkanes (C_8_–C_24_). SD: steam distillation; MSD: microwave-assisted steam distillation; RT: retention time; -: not detected.*

**Table 2:** Top-ranked *Corymbia citriodora* essential oil constituents docked against human COX-2 (PDB: 5KIR), sorted by binding affinity.

*Rank*	Compound	Binding Affinity (kcal·mol^−1^)	PubChem CID	Chemical Class
*1*	(Z,Z,Z)-1,5,9,9-Tetramethyl-1,4,7-cycloundecatriene	−7.9	5368784	Hydrocarbon sesquiterpene
*2*	Cardenolide	−7.9	53957771	Oxygenated monoterpene
*3*	Germacrene D	−7.4	5317570	Hydrocarbon sesquiterpene
*4*	2-Decyldecahydronaphthalene	−7.4	586218	Other
*5*	Citronellyl isovalerate	−7.1	111440	Oxygenated monoterpene
*6*	Guaiol	−7.1	227829	Oxygenated sesquiterpene
*7*	Farnesol (isomer)	−7.0	445070	Oxygenated sesquiterpene
*8*	β-Caryophyllene	−7.0	5281515	Hydrocarbon sesquiterpene
*9*	3-Methyl-2-(2-pentenyl)-2-cyclopenten-1-one	−6.7	1549018	Other
*10*	Geranyl acetate	−6.7	1549026	Oxygenated monoterpene
*11*	Isopulegol	−6.6	170833	Oxygenated monoterpene
*12*	α-Terpineol	−6.6	17100	Oxygenated monoterpene
*13*	Ledol	−6.6	11074994	Oxygenated sesquiterpene
*14*	Citronellal	−6.0	7794	Oxygenated monoterpene
*15*	Citronellol	−6.0	8842	Oxygenated monoterpene

**Table 3:** Molecular docking scores and conserved binding-site interactions of selected compounds with the 5KIR target protein.

Ligand	Binding Affinity	Interacting Residues	Common Residues with Co-Crystal
5KIR-Rofecoxib (Co-crystal)	−10.2	HIS B:90, GLN B:192, VAL B:349, LEU B:352, SER B:353, TYR B:355, TRP B:387, ARG B:513, ALA B:516, ILE B:517, PHE B:518, MET B:522, VAL B:523, GLY B:526, ALA B:527, SER B:530, LEU B:531	
5KIR-(Z,Z,Z)-1,5,9,9-Tetramethyl-1,4,7-cycloundecatriene	−8	SER B:353, TRP B:387, PHE B:518, LEU B:384, VAL B:523, MET B:522, GLY B:526, LEU B:531, VAL B:349, LEU B:352, ALA B:527, SER B:530.	SER B:353, TRP B:387, PHE B:518, VAL B:523, MET B:522, GLY B:526, LEU B:531, VAL B:349, LEU B:352, ALA B:527, SER B:530.
5KIR-2-Decyldecahydronaphthalene	−7.6	PHE B:518, LEU B:352, LEU B:531, MET B:113, LEU B:359, ALA B:527, TYR B:355, VAL B:116, TYR B:385, SER B:530, VAL B:349, VAL B:523, LEU B:384, TRP B:387, GLY B:526, MET B:522, SER B:353, ARG B:120.	VAL B:349, LEU B:352, SER B:353, TYR B:355, TRP B:387, PHE B:518, MET B:522, VAL B:523, GLY B:526, ALA B:527, SER B:530, LEU B:531.
5KIR-Citronellyl isovalerate	−7.2	VAL B:116, TYR B:355, SER B:353, ARG B:120, LEU B:359, VAL B:349, LEU B:531, LEU B:352, ALA B:527, GLY B:526, TYR B:385, TRP B:387, MET B:522, PHE B:518, VAL B:523, LEU B:384, PHE B:381, SER B:530, MET B:113.	VAL B:349, LEU B:352, SER B:353, TYR B:355, TRP B:387, PHE B:518, MET B:522, VAL B:523, GLY B:526, ALA B:527, SER B:530, LEU B:531.
5KIR-Germacrene D	−7.2	ARG B:513, HIS B:90, LEU B:352, SER B:353, TYR B:355, VAL B:523, PHE B:518, ARG B:120, VAL B:349, ALA B:527, GLY B:526, SER B:530, LEU B:531.	HIS B:90, VAL B:349, LEU B:352, SER B:353, TYR B:355, ARG B:513, PHE B:518, VAL B:523, GLY B:526, ALA B:527, SER B:530, LEU B:531.
5KIR-Cardenolide	−2.8	SER B:353, ARG B:120, LEU B:352, TRP B:387, LEU B:384, VAL B:349, SER B:530, MET B:522, GLY B:526, PHE B:518, VAL B:523, ALA B:527, TYR B:355, MET B:113, VAL B:116, LEU B:359, LEU B:531.	SER B:353, LEU B:352, TYR B:355, TRP B:387, VAL B:349, PHE B:518, MET B:522, VAL B:523, GLY B:526, ALA B:527, SER B:530, LEU B:531.

**Table 4:** Molecular Docking Scores and Conserved Binding-Site Residues of Selected Ligands Compared with the 3LN1–Celecoxib Co-crystal Complex.

Ligand	Binding Affinity	Interacting Residues	Common Residues with Co-Crystal
3LN1-Celecoxib (Co-crystal)	−12.2	MET A:508, TYR A:371, PHE A:367, SER A:516, LEU A:370, TRP A:373, GLY A:512, LEU A:345, VAL A:335, ALA A:513, LEU A:517, ILE A:503, PHE A:504, HIS A:75, ALA A:502, THR A:79, GLN A:178, LEU A:338, ARG A:499, SER A:339, GLY A:340, VAL A:102, ARG A:106, VAL A:509, TYR A:341.	-
3LN1 -(Z,Z,Z)-1,5,9,9-Tetramethyl-1,4,7-cycloundecatriene	−8.2	SER A:339, TRP A:373, MET A:508, PHE A:504, LEU A:370, VAL A:335, LEU A:338, ALA A:513, GLY A:512, SER A:516, TYR A:371, VAL A:509, LEU A:517	SER A:339, TRP A:373, MET A:508, PHE A:504, LEU A:370, VAL A:335, LEU A:338, ALA A:513, GLY A:512, SER A:516, TYR A:371, VAL A:509, LEU A:517
3LN1-2-Decyldecahydronaphthalene	−7.8	TYR A:341, HIS A:75, MET A:508, PHE A:504, TYR A:371, ALA A:513, TYR A:334, LEU A:338, VAL A:509, TRP A:373, SER A:339, VAL A:335, SER A:516, GLN A:178, ARG A:499, GLY A:512, ILE A:503, LEU A:370, PHE A:367	TYR A:341, HIS A:75, MET A:508, PHE A:504, TYR A:371, ALA A:513, LEU A:338, VAL A:509, TRP A:373, SER A:339, VAL A:335, SER A:516, GLN A:178, ARG A:499, GLY A:512, ILE A:503, LEU A:370, PHE A:367
3LN1-Citronellyl isovalerate	−7.2	MET A:99, VAL A:102, ARG A:106, VAL A:335, LEU A:338, SER A:339, TYR A:341, LEU A:345, PHE A:367, LEU A:370, TYR A:371, TRP A:373, PHE A:504, MET A:508, VAL A:509, GLY A:512, ALA A:513, and SER A:516.	VAL A:102, ARG A:106, VAL A:335, LEU A:338, SER A:339, TYR A:341, LEU A:345, PHE A:367, LEU A:370, TYR A:371, TRP A:373, PHE A:504, MET A:508, VAL A:509, GLY A:512, ALA A:513, and SER A:516.
3LN1-Germacrene D	−7.7	HIS A:75, ALA A:502, PHE A:504, ARG A:499, SER A:339, VAL A:509, TYR A:341, LEU A:338, VAL A:335, ARG A:106, ALA A:513, GLY A:512, LEU A:517, SER A:516	HIS A:75, ALA A:502, PHE A:504, ARG A:499, SER A:339, VAL A:509, TYR A:341, LEU A:338, VAL A:335, ARG A:106, ALA A:513, GLY A:512, LEU A:517, and SER A:516
3LN1-Cardenolide	−6.4	LEU A:517, ARG A:106, SER A:339, VAL A:335, PHE A:504, VAL A:509, TYR A:341, VAL A:102, TRP A:373, LEU A:338, GLY A:512, LEU A:78, PHE A:343, LEU A:345, ALA A:513, SER A:516, TYR A:371.	TYR A:371, SER A:516, TRP A:373, GLY A:512, LEU A:345, VAL A:335, ALA A:513, LEU A:517, PHE A:504, LEU A:338, SER A:339, VAL A:102, ARG A:106, VAL A:509, and TYR A:341

**Table 5:** Molecular docking scores and interacting residues of selected ligands with 4COX compared with the indomethacin co-crystal complex.

Ligand	Binding Affinity	Interacting Residues	Common Residues with Co-Crystal
4COX-Indomethacin (Co-crystal)	−9.6	SER A:530, ARG A:120, LEU A:531, VAL A:116, LEU A:359, ALA A:527, VAL A:349, HIS A:90, TYR A:355, ARG A:513, SER A:353, TYR A:385, GLY A:526, PHE A:518, TRP A:387, LEU A:384, PHE A:381, MET A:522, VAL A:523, LEU A:352.	-
4COX -(Z,Z,Z)-1,5,9,9-Tetramethyl-1,4,7-cycloundecatriene	−8.8	SER A:353, PHE A:518, TRP A:387, LEU A:384, MET A:522, VAL A:523, LEU A:352, ALA A:527, GLY A:526, SER A:530, VAL A:349, LEU A:531, TYR A:385, TYR A:348, PHE A:381.	SER A:530, LEU A:531, ALA A:527, VAL A:349, SER A:353, TYR A:385, GLY A:526, PHE A:518, TRP A:387, LEU A:384, PHE A:381, MET A:522, VAL A:523, LEU A:352.
4COX-2-Decyldecahydronaphthalene	−8.5	SER A:530, TYR A:355, LEU A:352, SER A:353, LEU A:531, VAL A:116, VAL A:349, ALA A:527, ARG A:120, VAL A:523, LEU A:359, TRP A:387, PHE A:381, GLY A:526, LEU A:384, TYR A:385, MET A:522, PHE A:518.	SER A:530, ARG A:120, LEU A:531, VAL A:116, LEU A:359, ALA A:527, VAL A:349, TYR A:355, SER A:353, TYR A:385, GLY A:526, PHE A:518, TRP A:387, LEU A:384, PHE A:381, MET A:522, VAL A:523, LEU A:352.
4COX-Citronellyl isovalerate	−7.6	VAL A:349, ARG A:120, SER A:353, LEU A:352, SER A:530, LEU A:359, TYR A:355, VAL A:523, GLY A:526, ALA A:527, LEU A:531, VAL A:116, LEU A:384, TRP A:387, PHE A:518, MET A:522, TYR A:385, PHE A:381.	SER A:530, ARG A:120, LEU A:531, VAL A:116, LEU A:359, ALA A:527, VAL A:349, TYR A:355, SER A:353, TYR A:385, GLY A:526, PHE A:518, TRP A:387, LEU A:384, PHE A:381, MET A:522, VAL A:523, LEU A:352.
4COX-Germacrene D	−8.3	ARG A:120, TYR A:355, SER A:353, VAL A:523, PHE A:518, LEU A:352, VAL A:349, MET A:522, GLY A:526, TRP A:387, ALA A:527, SER A:530, TYR A:348, PHE A:381, TYR A:385.	ARG A:120, TYR A:355, SER A:353, VAL A:523, PHE A:518, LEU A:352, VAL A:349, MET A:522, GLY A:526, TRP A:387, ALA A:527, SER A:530, PHE A:381, TYR A:385.
4COX-Cardenolide	−6.7	TYR A:348, SER A:353, VAL A:523, ARG A:120, ALA A:527, TYR A:355, TYR A:385, LEU A:352, PHE A:381, TRP A:387, LEU A:359, MET A:113, VAL A:116, LEU A:531, VAL A:349, SER A:530, GLY A:526, LEU A:384, MET A:522, PHE A:518.	SER A:353, VAL A:523, ARG A:120, ALA A:527, TYR A:355, TYR A:385, LEU A:352, PHE A:381, TRP A:387, LEU A:359, VAL A:116, LEU A:531, VAL A:349, SER A:530, GLY A:526, LEU A:384, MET A:522, PHE A:518.

**Table 6:** Molecular Docking Scores and Conserved Binding-Site Residues of Selected Ligands Compared with the 3E7G–AR-C95791 Co-crystal Complex.

Ligand	Binding Affinity	Interacting Residues	Common Residues with Co-Crystal
3NL1-AR-C95791 (Co-crystal)	−8.4	MET A:434, MET A:374, CYS A:200, GLY A:371, TRP A:372, ILE A:201, ALA A:439, TYR A:489, PHE A:369, LEU A:209, TRP A:194, GLY A:202, TYR A:373, ILE A:244	-
3NL1 -(Z,Z,Z)-1,5,9,9-Tetramethyl-1,4,7-cycloundecatriene	−6.8	GLY A:371, ASN A:370, MET A:355, VAL A:352, TRP A:194, PHE A:369, CYS A:200	CYS A:200, GLY A:371, PHE A:369, TRP A:194
3NL1-2-Decyldecahydronaphthalene	−8.2	TRP A:194, TRP A:372, CYS A:200, SER A:242, GLN A:205, TYR A:489, ILE A:244, ASN A:370, GLU A:377, ILE A:201, GLY A:202, MET A:374, MET A:434, LEU A:209, GLY A:371, PHE A:369	TRP A:194, TRP A:372, CYS A:200, TYR A:489, ILE A:244, ILE A:201, GLY A:202, MET A:374, MET A:434, LEU A:209, GLY A:371, PHE A:369
3NL1-Citronellyl isovalerate	−6.9	ASN A:370, GLY A:202, MET A:355, GLY A:371, ILE A:201, SER A:242, ALA A:197, PHE A:369, CYS A:200, ILE A:244, TRP A:194, TYR A:489, LEU A:209	CYS A:200, GLY A:371, ILE A:201, TYR A:489, PHE A:369, LEU A:209, TRP A:194, GLY A:202, ILE A:244
3NL1-Germacrene D	−7.2	GLN A:205, SER A:242, GLY A:202, TRP A:194, TRP A:372, GLY A:371, ASN A:370, PHE A:369, PRO A:350, CYS A:200	CYS A:200, GLY A:371, TRP A:372, TRP A:194, GLY A:202, PHE A:369
3NL1-Cardenolide	−8.8	PRO A:350, ILE A:201, TRP A:372, GLU A:377, LEU A:209, PHE A:369, MET A:374, ASN A:370, SER A:242, CYS A:200, ARG A:199, GLY A:371, VAL A:352, TRP A:463, TRP A:194	MET A:374, CYS A:200, GLY A:371, TRP A:372, ILE A:201, PHE A:369, LEU A:209, TRP A:194

**Table 7:** Global quantum chemical descriptors calculated at the B3LYP/3-21G/CPCM level for the top five candidate compounds.

Parameter	Unit	(Z,Z,Z)-1,5,9,9-Tetramethyl-1,4,7-cycloundecatriene	2-Decyldecahydronaphthalene	Citronellyl Isovalerate	Germacrene D	Cardenolide
HOMO energy (E_HOMO)	eV	−5.62	−7.2518	−6.2173	−5.4766	−7.0820
LUMO energy (E_LUMO)	eV	0.50	+2.9982	+0.1886	−0.0125	−0.9027
HOMO–LUMO gap (ΔE)	eV	6.12	10.2500	6.4058	5.4640	6.1793
Ionization potential (I)	eV	5.62	7.2518	6.2173	5.4766	7.0820
Electron affinity (A)	eV	−0.50	−2.9982	−0.1886	0.0125	0.9027
Electronegativity (χ)	eV	2.56	2.1268	3.0143	2.7445	3.9924
Chemical potential (μ)	eV	−2.56	−2.1268	−3.0143	−2.7445	−3.9924
Chemical hardness (η)	eV	3.06	5.1250	3.2029	2.7320	3.0897
Softness (S)	eV^−1^	0.163	0.09756	0.1561	0.1830	0.16183
Electrophilicity index (ω)	eV	1.07	0.4413	1.4184	1.3786	2.5784
Dipole moment	Debye	0.6929	0.0296	2.0809	0.9166	7.1675

**Table 8:** Summary of binding free energies and dominant stabilizing residues for the (Z,Z,Z)-1,5,9,9-tetramethyl-1,4,7-cycloundecatriene–COX-2 complex.

Method	ΔG_bind (kcal·mol^−1^)	Dominant Stabilizing Residues
AutoDock Vina **v1.1.2** (docking)	−8.0	Val349, Leu352, Leu384, Trp387, Met522, Val523, Ala527
MM/GBSA	−23.98	Val349, Leu352, Ser353, Val523, Gly526, Ala527, Ser530
MM/PBSA	−21.95	Val349, Leu352, Val523, Ala527, Leu531

**Table 9:** In silico ADMET profile of the five candidate compounds predicted with the pkCSM platform.

Compartment	Property/Model	Unit	1	2	3	4	5
Absorption	Water solubility	log mol·L^−1^	−5.373	−5.166	−7.780	−5.682	−4.839
	Caco-2 permeability	log Papp (10^−6^ cm·s^−1^)	1.266	1.203	1.391	1.436	1.541
	Intestinal absorption (human)	% absorbed	96.611	100.000	92.404	95.590	94.624
	Skin permeability	log Kp (cm·h^−1^)	−1.555	−2.702	−2.668	−1.429	−1.784
	P-glycoprotein substrate	Yes/No	Yes	No	No	No	No
	P-glycoprotein I inhibitor	Yes/No	No	Yes	No	No	No
	P-glycoprotein II inhibitor	Yes/No	No	No	Yes	No	No
Distribution	VDss (human)	log L·kg^−1^	0.629	−0.184	0.427	0.544	0.221
	Fraction unbound (human)	Fu	0.306	0.007	0.000	0.261	0.205
	BBB permeability	log BB	0.492	0.740	0.966	0.723	0.640
	CNS permeability	log PS	−2.181	−1.443	−0.884	−2.138	−1.897
Metabolism	CYP2D6 substrate	Yes/No	No	No	No	No	No
	CYP3A4 substrate	Yes/No	No	Yes	Yes	No	No
	CYP1A2 inhibitor	Yes/No	Yes	No	Yes	No	No
	CYP2C19 inhibitor	Yes/No	No	No	No	No	No
	CYP2C9 inhibitor	Yes/No	No	No	No	No	No
	CYP2D6 inhibitor	Yes/No	No	No	No	No	No
	CYP3A4 inhibitor	Yes/No	No	No	No	No	No
Excretion	Total clearance	log mL·min^−1^·kg^−1^	1.088	0.519	1.395	1.420	1.491
	Renal OCT2 substrate	Yes/No	No	No	No	No	No
Toxicity	AMES toxicity	Yes/No	No	No	No	No	No
	Maximum tolerated dose (human)	log mg·kg^−1^·day^−1^	0.987	−0.376	−0.528	0.497	0.625
	hERG I inhibitor	Yes/No	No	No	No	No	No
	hERG II inhibitor	Yes/No	No	No	Yes	No	No
	Oral rat acute toxicity (LD50)	mol·kg^−1^	2.316	2.489	1.874	1.634	1.654
	Oral rat chronic toxicity (LOAEL)	log mg·kg^−1^·day^−1^	1.258	2.145	1.359	1.413	2.471
	Hepatotoxicity	Yes/No	No	No	No	No	No
	Skin sensitization	Yes/No	Yes	No	Yes	Yes	Yes
	*T. pyriformis* toxicity	log µg·L^−1^	1.314	0.330	0.905	1.671	2.228
	Minnow toxicity	log mM	0.674	−2.459	−1.691	0.257	−0.421

Compounds: 1, (Z,Z,Z)-1,5,9,9-tetramethyl-1,4,7-cycloundecatriene; 2, cardenolide; 3, germacrene D; 4, 2-decyldecahydronaphthalene; 5, citronellyl isovalerate. AMES, Ames mutagenicity test; BBB, blood–brain barrier; Caco-2, human colorectal adenocarcinoma cell line; CNS, central nervous system; CYP, cytochrome P450; Fu, fraction unbound; hERG, human ether-à-go-go-related gene; LD50, median lethal dose; LOAEL, lowest-observed-adverse-effect level; OCT2, organic cation transporter 2; Papp, apparent permeability coefficient; VDss, steady-state volume of distribution.

## Data Availability

The original contributions presented in this study are included in the article. Further inquiries can be directed to the corresponding author.
